# Identification of a common ketohexokinase-dependent link driving alcohol intake and alcohol-associated liver disease in mice

**DOI:** 10.1038/s42255-025-01402-x

**Published:** 2025-11-10

**Authors:** Ana Andres-Hernando, David J. Orlicky, Gabriela E. Garcia, Esteban C. Loetz, Richard Montoya, Vijay Kumar, Devin P. Effinger, Masanari Kuwabara, So Young Bae, Laura Lorenzo-Rebenaque, Elena Fauste, Richard L. Bell, Nicholas Grahame, Suthat Liangpunsakul, Hahn Kim, Sundeep Dugar, Paul Maffuid, Takahiko Nakagawa, Michael F. Wempe, J. Mark Petrash, Dean R. Tolan, Sondra T. Bland, Richard J. Johnson, Miguel A. Lanaspa

**Affiliations:** 1 Division of Endocrinology, Metabolism and Diabetes, University of Colorado Denver, Aurora, CO USA; 2 Department of Pathology, University of Colorado School of Medicine, Aurora, CO USA; 3 Division of Renal Diseases and Hypertension, University of Colorado Denver, Aurora, CO USA; 4 Department of Psychology, University of Colorado Denver, Aurora, CO USA; 5 Skaggs School of Pharmacy and Pharmaceutical Sciences, Aurora, CO USA; 6 Department of Psychiatry, University of Colorado School of Medicine, Aurora, CO USA; 7 Department of Biology, Boston University, Boston, MA USA; 8 Institute of Science and Animal Technology, Universitat Politècnica de Valencia, Valencia, Spain; 9 Facultad de Farmacia, Universidad San Pablo-CEU, CEU Universities, Montepríncipe, Boadilla del Monte, Madrid, Spain; 10 Department of Psychiatry, Indiana University Indianapolis, Indianapolis, IN USA; 11 Department of Psychology, Indiana University Indianapolis, Indianapolis, IN USA; 12 Division of Gastroenterology and Hepatology, Department of Internal Medicine, Indiana University School of Medicine, Indianapolis, IN USA; 13 Department of Biochemistry and Molecular Biology, Indiana University School of Medicine, Indianapolis, IN USA; 14 Roudebush Veterans’ Administration Medical Center, Indianapolis, IN USA; 15 Frick Laboratory, Department of Chemistry, Princeton, NJ USA; 16 Aayam Therapeutics, San Jose, CA USA; 17 Department of Nephrology, Rakuwakai Otowa Hospital, Kyoto, Japan; 18 Department of Biological and Physical Sciences, Kentucky State University, Frankfort, KY USA; 19 Department of Ophthalmology, University of Colorado School of Medicine, Aurora, CO USA

**Keywords:** Metabolic disorders, Metabolic diseases, Metabolism

## Abstract

Alcohol and sugar share reinforcing properties and both contribute to liver disease progression, ultimately leading to cirrhosis. Emerging evidence suggests that ethanol activates the aldose reductase pathway, resulting in endogenous fructose production. Here we investigated whether alcohol preference and alcohol-associated liver disease (ALD) are mediated through fructose metabolism by ketohexokinase (KHK)-A/C. Using global, conditional and tissue-specific KHK-A/C knockout mice, we assessed ethanol intake, reinforcement behaviours and liver injury. Ethanol consumption increased portal vein osmolality and activated the polyol pathway in the liver and intestine, leading to fructose production metabolized by KHK-A/C. Mice lacking KHK-A/C showed reduced ethanol preference across multiple paradigms, including two-bottle choice, conditioned place preference and operant self-administration, alongside decreased ∆FosB expression in the nucleus accumbens. Both genetic deletion and pharmacologic inhibition of KHK-A/C suppressed ethanol intake. Hepatocyte-specific KHK-A/C knockout mice displayed partially reduced alcohol consumption, potentially linked to altered aldehyde dehydrogenase expression, while intestinal KHK-A/C deletion restored glucagon-like peptide-1 levels—a hormone known to suppress alcohol intake. Under ethanol pair-matched conditions, global and liver-specific KHK-A/C knockout mice were protected from ALD, with marked reductions in hepatic steatosis, inflammation and fibrosis. These findings identify ethanol-induced fructose metabolism as a key driver of excessive alcohol consumption and ALD pathogenesis. Given that ALD and metabolic dysfunction-associated steatotic liver disease share fructose-dependent mechanisms, targeting fructose metabolism may offer a novel therapeutic approach for treating alcohol use disorder and related liver injury.

## Abstract (web summary)

Ethanol-induced fructose metabolism, mediated by KHK-A/C, drives excessive alcohol consumption and pathogenesis of alcoholic liver disease.

## Main

Sugar and alcohol may share common mechanistic pathways underlying preference and reinforcement, leading some to refer to sugar as ‘alcohol without the buzz’^1^. Individuals with alcohol use disorder (AUD) exhibit an increased preference for sugar^2^, a trait that is also observed in their children^3^. Similarly, laboratory rats that are given access to ethanol consume more sugar, while those consuming sugar also drink more ethanol^4^. These bidirectional effects highlight the reinforcing properties of both substances. Sugar, whether in the form of sucrose or high fructose corn syrup is composed of glucose and fructose. Of note, the fructose component in sugar is highly reinforcing in both mice and rats, stimulating dopamine release^5^ and inducing ∆FosB expression^6^ in the nucleus accumbens, which are key neurochemical responses also triggered by ethanol^7^. ∆FosB is a transcription factor associated with addiction-related activation of the mesocorticolimbic reward pathway^8^. Intermittent access to sugar can lead to binge-like feeding behaviour in mice, and withdrawal symptoms upon sugar deprivation^5^, paralleling withdrawal syndromes that are observed with ethanol^9^. We have recently demonstrated that intake and appetite for fructose persist in ‘taste-blind’ mice, indicating that preference is not solely driven by taste perception but rather is also regulated by its metabolic processing^6,10^.

Other important parallels between sugar and ethanol include their capacity to induce liver disease. Sugar—particularly its fructose component—is now recognized as a major contributor to metabolic-associated steatotic liver disease (MASLD)^11^. Notably, both ALD and MASLD share similar histopathological features, including steatohepatitis and cirrhosis^12^. In animal studies, co-administration of fructose and ethanol using pair-feeding models results in greater expression of liver disease markers than ethanol alone^13^. Moreover, ethanol and fructose trigger comparable metabolic responses in the liver, including purine nucleotide turnover, inhibition of adenosine monophosphate-activated protein kinase, activation of nicotinamide adenine dinucleotide phosphate oxidase, mitochondrial oxidative stress and several other pathological processes^11^.

Our group has demonstrated that, in addition to dietary intake, fructose can be produced endogenously through activation of aldose reductase (AR) and the polyol pathway. The metabolism of this endogenously generated fructose via KHK-A/C has a critical pathogenic role in the development of fatty liver disease^14^, as well as in liver and kidney injury^15^. Recent studies suggest that ethanol may also induce endogenous fructose production, potentially contributing to the pathogenesis of ALD. Alcohol ingestion increases serum osmolality^16^, which can upregulate AR expression^17^. Although AR is not typically expressed in the healthy human liver, it is detected in the livers of individuals with ALD^18,19^. Wang et al. found that fructose is present in the livers of both humans and rats with ALD^19^, and that AR-deficient animals are protected from ALD^19,20^. Moreover, ethanol-induced lipogenesis in human hepatocytes (HepG2 cells) is blocked by AR inhibition^21^. On the basis of these findings, we hypothesized that fructose metabolism is a critical mechanism through which ethanol drives preference, reinforcement and liver injury.

## Results

### Preference for ethanol is dependent on fructose metabolism

**Fig. 1:**
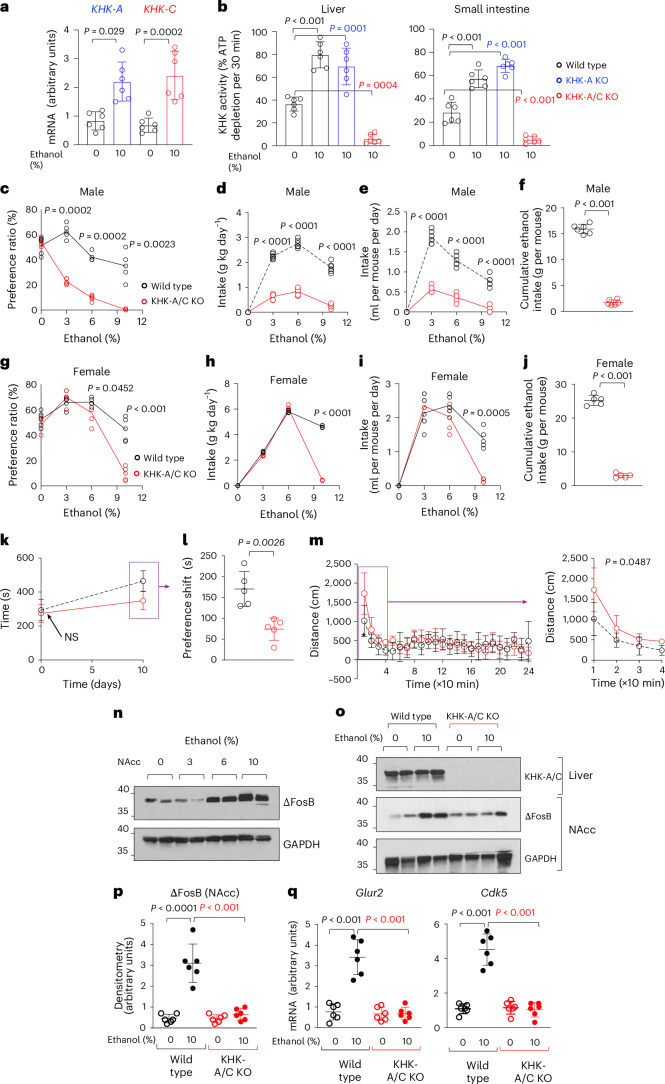
Metabolism of endogenous fructose via KHK promotes ethanol intake and preference in mice. **a**, mRNA expression of *KHK-A* and *KHK-C* isoforms in the liver of wild-type (mice control (0%) or chronically exposed to ethanol (10%). **b**, KHK activity in the liver and intestine of wild-type, KHK-A KO and KHK-A/C KO mice control (0%) or chronically exposed to ethanol (10%). **c**, Ethanol-water two-bottle choice preference ratio of male wild-type (black) and KHK-A/C KO (red) mice at three different ethanol concentrations (vol/vol 3%, 6% and 10%). **d**, Average ethanol intake in g kg day^−1^ in the same groups as in **c**. **e**, Average ethanol intake in ml per mouse per day in the same groups as in **c**. **f**, Cumulative 30-week ethanol intake in wild-type (black) and KHK-A/C KO (red) mice on ethanol-water two-bottle choice preference paradigms. **g**, Ethanol-water two-bottle choice preference ratio of female wild-type (black) and KHK-A/C KO (red) mice at three different ethanol concentrations (vol/vol 3%, 6% and 10%). **h**, Average ethanol intake in g kg day^−1^ in the same groups as in **g**. **i**, Average ethanol intake in ml per mouse per day in the same groups as in **g**. **j**, Cumulative 30-week ethanol intake in wild-type (black) and KHK-A/C KO (red) mice on ethanol-water two-bottle choice preference paradigms. **k**, Total time spent in ethanol-conditioned chamber at days 0 and 10 after conditioning in wild-type (black) and KHK-A/C KO (red) mice injected with 2 g kg^−1^ ethanol. **l**, Preference shift to ethanol-conditioned chamber (CPP) of wild-type (black) and KHK-A/C KO (red) mice injected with 2 g kg^−1^ ethanol. **m**, Locomotor activity of wild-type (black) and KHK-A/C KO (red) mice injected with 2 g kg^−1^ ethanol and recorded in 10-min intervals analysed for 240 min (left) or the first 40 min (right). **n**, Representative western blot for ∆FosB and GAPDH loading control in nucleus accumbens (NAcc) of wild-type mice exposed via two-bottle choice preference to increasing concentrations of ethanol for 30 weeks. **o**,**p**, Representative western blot (**o**) and densitometry (**p**) for KHK-A/C (liver), ∆FosB and GAPDH loading control in nucleus accumbens of wild-type and KHK-A/C KO mice exposed to water control (0% ethanol) or 10% ethanol via two-bottle choice preference for 30 weeks. **q**, mRNA expression of ∆FosB target genes (*Glur2* and *Cdk5*) in nucleus accumbens of wild-type and KHK-A/C KO mice exposed to water control (0% ethanol) or 10% ethanol via two-bottle choice preference for 30 weeks. Data in **a**–**l**,**p**,**q** are shown as individual values. Mean ± s.d. additionally presented in **a**,**b**,**f**,**j**. Data for **a**,**b**,**p**,**q** were analysed using one-way ANOVA followed by Tukey’s post hoc multiple comparison test. Data for **c**–**m** were analysed using a two-tailed paired *t*-test at each timepoint. Data in **a**–**j**,**p**,**q** were obtained from two separate studies, and the study for **k**–**m** was conducted once. Sample size: *n* = 5–6 mice per group. See also Extended Data Figs. 1–4. NS, not significant. Source data

To investigate whether ethanol preference depends on fructose metabolism, we first assessed the expression of KHK-A/C isoforms. mRNA levels of both *KHK-A* and *KHK-C* isoforms were significantly increased in the livers of ethanol-conditioned mice (Fig. 1a), indicating the involvement of both isoforms, with no evidence of alternative splicing favouring one isoform over the other. Consistently, chronic ethanol exposure increased both KHK protein expression and enzymatic activity in the liver and intestine (Fig. 1b). As expected, ethanol-induced KHK activation was absent in systemic KHK-A/C knockout (KO) mice but remained intact in KHK-A isoform-specific KO (KHK-A KO) mice, suggesting that ethanol predominantly activates the KHK-C isoform.

**Extended Data Fig. 1:**
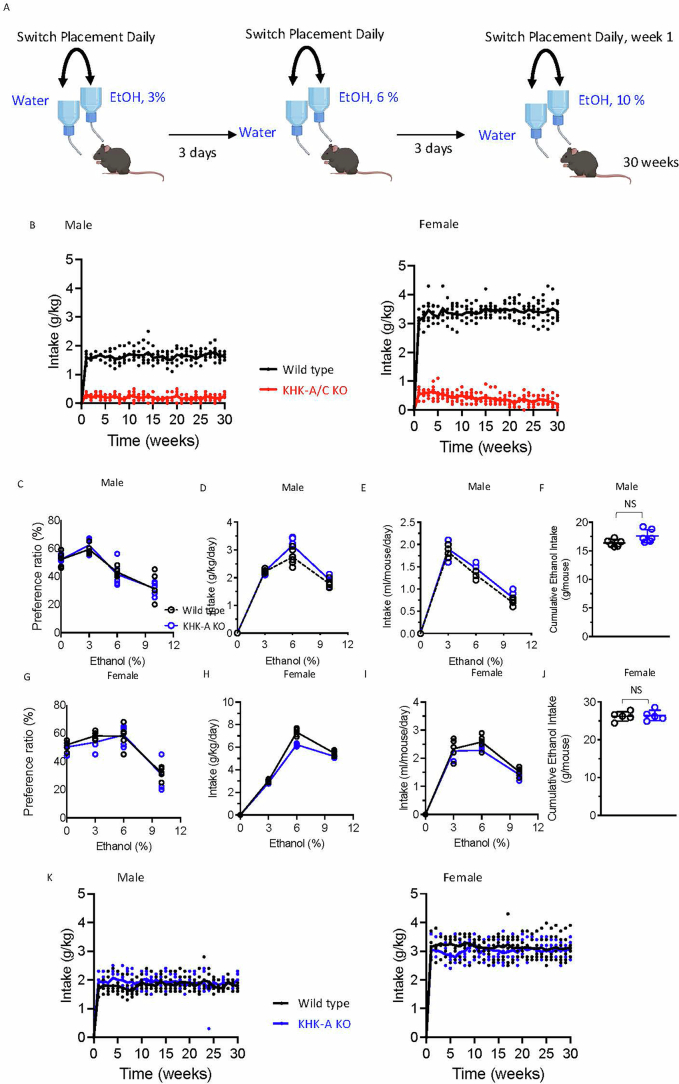
Accumulated ethanol intake and preference in wild type, KHK-A/C KO and KHK-A mice. **A**) proposed two-bottle choice preference paradigm to mice gradually exposed to escalating concentrations of ethanol. Mice choose between water or a 3% ethanol solution for 3 days, the amount of ethanol is gradually increased until 10%, bottle position was switched to prevent side-bias. Mice are then kept on the water and 10% ethanol solution for 30 weeks. **B**) 30-week ethanol intake in wild type (black) and KHK-A/C (red) male and female mice. **C**) Ethanol-water two-bottle choice preference ratio of male wild type (black) and KHK-A KO (blue) mice at three different ethanol concentrations (v/v 3 %, 6 % and 10%). **D**) Average ethanol intake in g/kg/day in the same groups as in **C**). **E**) Average ethanol intake in ml/mouse/day in the same groups as in **C**. **F**) Cumulative 30 weeks ethanol intake in wild-type (black) and KHK-A (blue) mice on ethanol-water two-bottle choice preference protocols. **G**) Ethanol-water two-bottle choice preference ratio of female wild type (black) and KHK-A KO (blue) mice at three different ethanol concentrations (v/v 3 %, 6 % and 10%). **H**) Average ethanol intake in g/kg/day in the same groups as in **G**). **I**) Average ethanol intake in ml/mouse/day in the same groups as in **G**. **J**) Cumulative 30 weeks ethanol intake in wild type (black) and KHK-A (blue) mice on ethanol-water two-bottle choice preference protocols. **K**) 30-week ethanol intake in wild type (black) and KHK-A (blue) male and female mice. The data are presented as individual values and analyzed by One-Way ANOVA with Tukey post hoc analysis. Data in panels F and J is presented as mean ± standard deviation (SD). NS=not significant. *n* = 6 mice per group. Panel **a** created with BioRender.com. Source data

To determine whether loss of KHK-A/C affects ethanol intake and/or preference, we used a two-bottle choice paradigm with escalating ethanol concentrations (experimental design shown in Extended Data Fig. 1a). As shown in Fig. 1c–j, KHK-A/C KO mice, which lack the ability to metabolize fructose, exhibited a significant reduction in both ethanol preference and intake. This effect was observed in both male (Fig. 1c–f) and female (Fig. 1g–j) mice and resulted in a markedly lower cumulative ethanol intake over a 30-week period (Fig. 1f,j and Extended Data Fig. 1b). By contrast, mice lacking only the KHK-A isoform displayed ethanol preference and consumption comparable to wild-type controls (Extended Data Fig. 1c–k), further supporting a predominant role for the KHK-C isoform in regulating ethanol intake.

To assess the rewarding properties of ethanol, we next performed a conditioned place preference (CPP) assay. Following ten conditioning sessions (five with saline and five with ethanol, 2 g kg^−1^), mice were tested in the absence of ethanol for chamber preference. Wild-type mice exhibited a strong CPP response, spending significantly more time in the ethanol-paired chamber. By contrast, KHK-A/C KO mice demonstrated a blunted shift in preference and spent less time in the conditioned chamber relative to the wild-type control condition (Fig. 1k–l), indicating reduced ethanol-associated reward.

**Extended Data Fig. 2:**
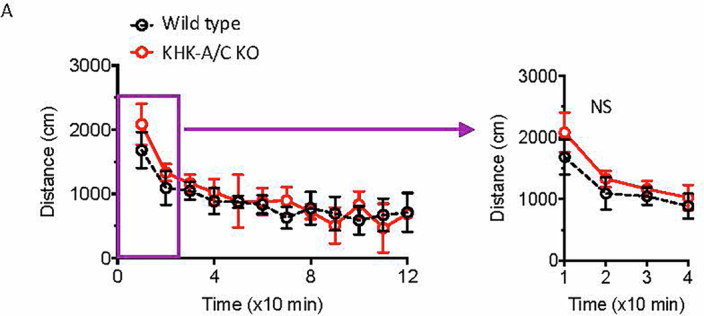
Locomotor activity in saline injected WT and KHK-A/C KO mice. **A**) Locomotor activity of wild type (black) and KHK-A/C KO (red) mice injected with 2 g/kg ethanol and recorded in 10-min intervals analyzed for 120 min (left) or the first 40 min (right). The data are presented as the mean ± SD and analyzed by One-Way ANOVA with Tukey post hoc analysis. NS: not significant. Source data

**Extended Data Fig. 3:**
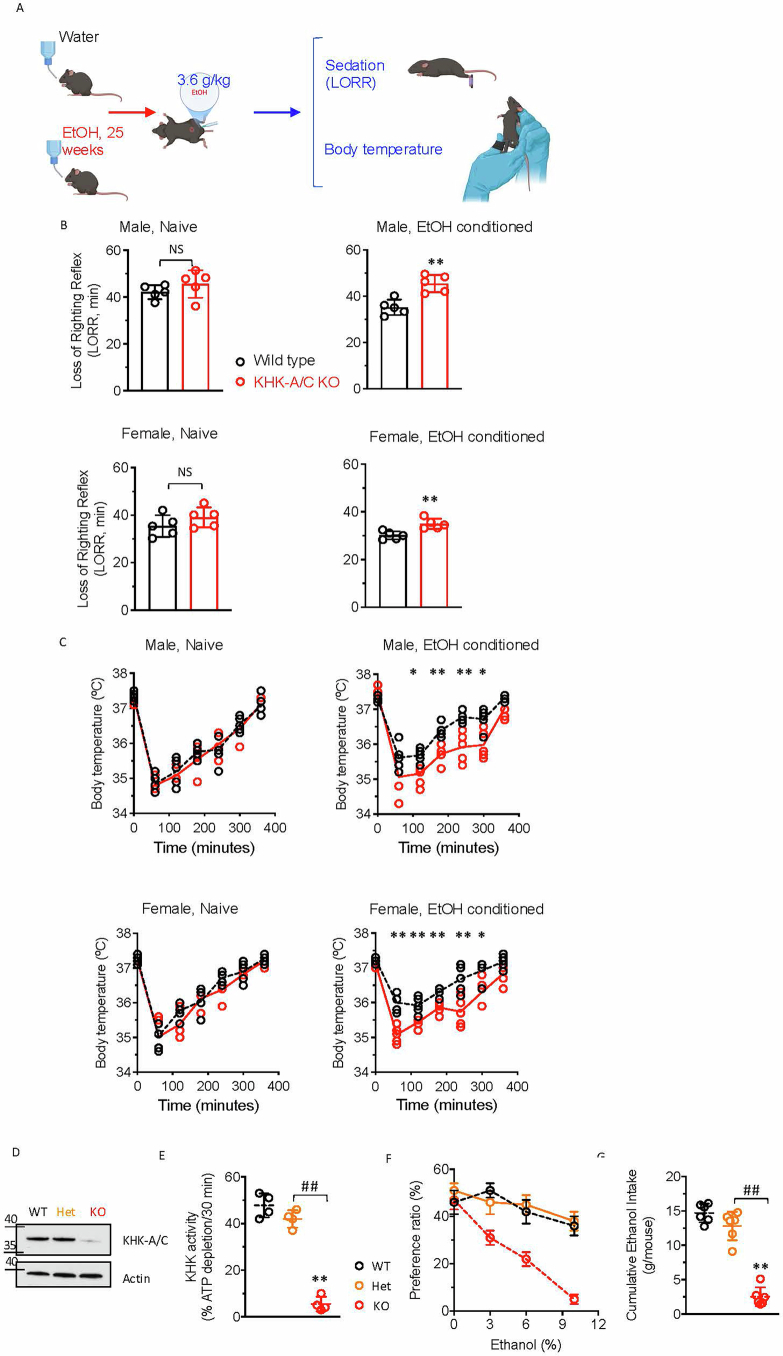
Effect of KHK deletion in ethanol-induced sedation and hypothermia. **A**) Wild type (WT, black) and KHK-A/C KO (red) mice were treated as water control or exposed to ethanol for 25 weeks. After the conditioning period, mice were injected with ethanol (3.6 g/kg) and sedation determined by LORR and body temperature. **B**) LORR in male and female mice control (naïve) and ethanol conditioned. **C**) Body temperature after the injection in male and female mice control (naïve) and ethanol conditioned. **D**) Representative western blot for liver KHK in wild type (WT), KHK-A/C heterozygous (Het, orange) and knockout (KO, red) mice and actin control. **E**) Hepatic KHK activity in wild type (WT), KHK-A/C heterozygous (Het, orange) and knockout (KO, red) mice. **F**) Ethanol-water two-bottle choice preference ratio of wild type (WT), KHK-A/C heterozygous (Het, orange) and knockout (KO, red) mice on increasing ethanol (0-10%) concentrations. **G**) 30-week cumulative ethanol intake wild type (WT), KHK-A/C heterozygous (Het, orange) and knockout (KO, red) mice. The data are presented as individual values and analyzed by 2-tail test (**B**) and 2-Way ANOVA (**C**) with Tukey post hoc multiple comparison analysis. Data in panels B, E-G is presented as mean ± standard deviation (SD). For **B-C**) *P < 0.05, **P < 0.01, NS=not significant versus WT. For **E-G**) **P < 0.01 versus WT, ## *P* < 0.01, NS=not significant. *n* = 5 (**B-C**) and *n* = 6 (**E**-**G**) mice per group. Panel **a** created with BioRender.com. Source data

In locomotor activity studies, no significant strain differences were observed in response to saline (Extended Data Fig. 2). However, following ethanol administration (2 g kg^−1^), KHK-A/C KO mice exhibited increased locomotor activity during the initial 10 min, suggesting an altered behavioural response (activation) that may reflect reduced reward sensitivity (Fig. 1m). We next examined the sedative and hypothermic effects of ethanol. At the dose used for the CPP and locomotor assays (2 g kg^−1^), ethanol did not induce significant sedation or loss of righting reflex (LORR). Therefore, a higher dose (3.6 g kg^−1^), consistent with previous studies^22^, was used to assess these outcomes. As shown in Extended Data Fig. 3a–c, acute ethanol administration elicited hypothermia and LORR in ethanol-naive wild-type and KHK-A/C KO mice. Chronic ethanol exposure attenuated these effects, particularly in wild-type mice. By contrast, ethanol-conditioned KHK-A/C KO mice exhibited significantly prolonged LORR recovery times (Extended Data Fig. 3b) and a more pronounced drop in body temperature (Extended Data Fig. 3c), indicating enhanced sensitivity to the sedative and hypothermic effects of ethanol, particularly at higher doses of, or after chronic exposure to, ethanol.

To explore the molecular effects of ethanol intake, we assessed expression levels of ∆FosB and its downstream targets, *Glur2*—a subunit of the AMPA receptor involved in synaptic plasticity and reinforcement behaviour^23^—and *Cdk5*—a critical kinase in postmitotic neurons that influences synaptic strength and plasticity^24^—in the nucleus accumbens. Ethanol-treated wild-type mice showed increased expression of all three genes, whereas KHK-A/C KO mice failed to exhibit similar changes (Fig. 1n–q). This suggests that either KHK-A/C mice consumed insufficient ethanol to trigger these neuroadaptive responses, or they are intrinsically less responsive to ethanol-induced molecular signalling, at least within the nucleus accumbens. Finally, we tested whether partial loss of KHK-A/C would result in a gene dosage-dependent phenotype. However, KHK expression and enzymatic activity in the liver of KHK-A/C heterozygous mice were comparable to wild-type controls (Extended Data Fig. 3d,e). Consistent with this, ethanol preference and intake in heterozygous mice did not differ from wild-type mice and did not show a dose-dependent effect (Extended Data Fig. 3f,g).

### Ethanol stimulates endogenous fructose production and metabolism

**Extended Data Fig. 4:**
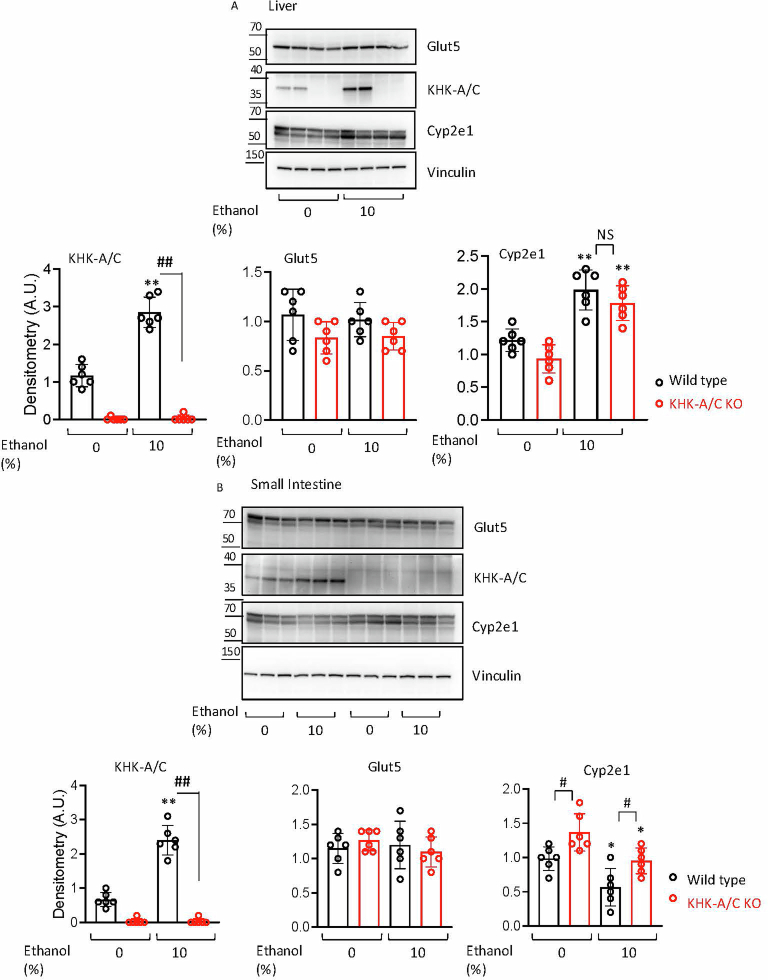
Effect of ethanol conditioning on fructose transport and Cyp2E1 expression in gut and liver. **A**) Representative western blot and densitometry for liver Glut5, Cyp2E1, and KHK-A/C in wild-type (WT, black) and KHK-A/C knockout (KO, red) mice. **B**) Representative western blot and densitometry for intestinal Glut5, Cyp2E1, and KHK-A/C in wild-type (WT, black) and KHK-A/C knockout (KO, red) mice. Data are shown as individual values and presented as mean ± standard deviation (SD). Panels **B**–**C** were analyzed by two-way ANOVA with Tukey’s post hoc multiple comparisons test. **P < 0.01 versus respective strain with 0% ethanol; #P < 0.05, ##P < 0.01; NS, not significant. Sample size: n = 6 mice per group. Source data

After establishing both KHK activation and altered behavioural responses to ethanol in systemic KHK-A/C KO mice, we next sought to identify the mechanism by which ethanol promotes fructose metabolism. Notably, expression of the primary fructose transporter, GLUT5, in the liver and small intestine remained unchanged following ethanol exposure (Extended Data Fig. 4a,b). This finding suggests that fructose accumulation is probably of endogenous origin rather than due to enhanced intestinal absorption and that ethanol-induced KHK activation may be driven by locally synthesized fructose.

**Fig. 2:**
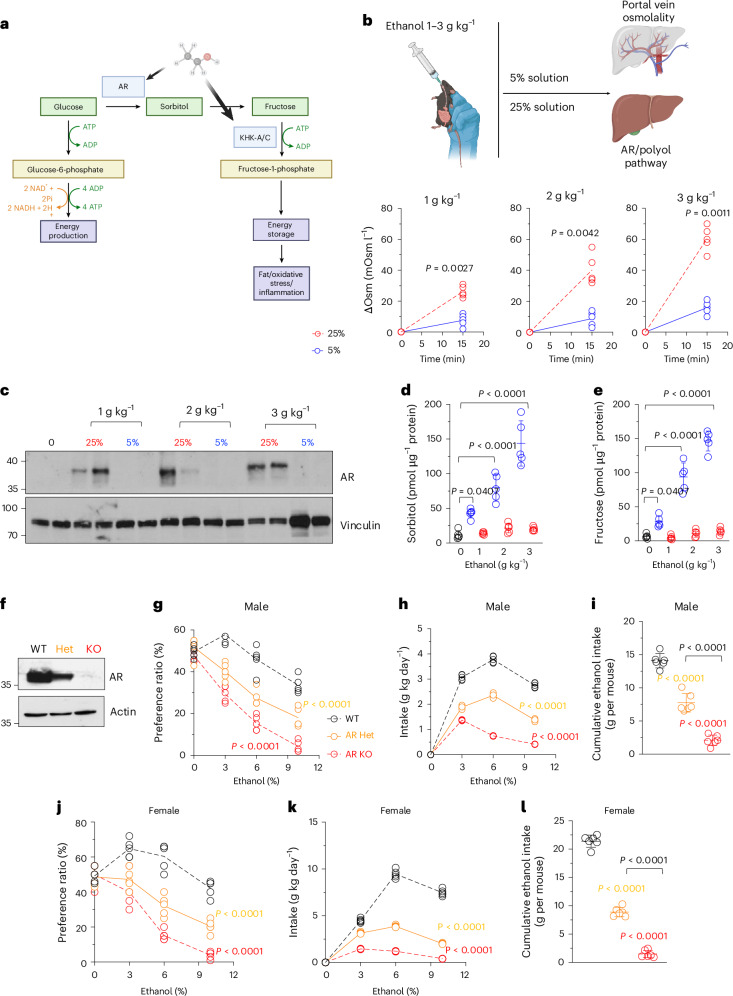
Phenotypic response of ethanol-mediated endogenous fructose production. **a**, Proposed mechanism for endogenous fructose production from glucose via activation of AR and the polyol pathway. In normal conditions, glucose is phosphorylated to glucose-6-phosphate, which is either used for glycogen production or glycolysis or used via the pentose phosphate route for energy production. Ethanol-dependent osmotic activation of AR and KHK-A/C shifts glucose use to produce sorbitol and fructose. In turn, fructose metabolism (fructolysis) via KHK-A/C promotes the store of energy as fat, causing oxidative stress and inflammation. **b**, Portal vein osmolality 15 min after receiving oral gavage of ethanol (1–3 g kg^−1^) either as a 5% or a 25% solution. **c**, Representative western blot and densitometry for liver AR and vinculin loading control in wild-type (WT) mice receiving an oral gavage of ethanol (1–3 g kg^−1^) as a concentrated (25%) or diluted (5%) solution for four consecutive days. **d**,**e**, Intrahepatic sorbitol (**d**) and fructose (**e**) at day 4 after oral gavage. **f**, Representative western blot for AR in the liver of ethanol-conditioned wild-type, heterozygous (AR Het) and knockout (AR KO) mice to show a dose-dependent expression of AR with the genotype. **g**, Ethanol-water two-bottle choice preference ratio of male wild-type (black), AR Het (orange) and AR KO (red) mice at three different ethanol concentrations (vol/vol 3%, 6% and 10%). **h**, Average ethanol intake in g kg day^−1^ in the same groups as in **g**. **i**, Cumulative 30-week ethanol intake. **j**, Ethanol-water two-bottle choice preference ratio of female wild-type (black), AR Het (orange) and AR KO (red) mice at three different ethanol concentrations (vol/vol 3%, 6% and 10%). **k**, Average ethanol intake in g kg day^−1^ in the same groups as in **i**. **l**, Cumulative 30-week ethanol intake. The data are shown as individual values and were analysed using either one-way ANOVA with Tukey’s post hoc test (**d**–**l**) or a paired two-sided *t*-test (**b**). Mean ± s.d. is additionally presented in **d**. Data were obtained from two separate studies and pooled together for statistical analysis. Sample size: *n* = 5–6 mice per group. Sample size: *n* = 5 mice per group. See also Extended Data Fig. 7. Panels **a** and **b** created with BioRender.com. Source data

Previous studies from our group and others have demonstrated that upregulation of AR initiates the polyol pathway, a metabolic cascade that converts glucose to sorbitol and subsequently to fructose, thereby generating endogenous fructose (Fig. 2a). A well-established trigger for AR activation is elevated osmolality. Given that ethanol is a hyperosmolar compound, we hypothesized that its osmotic properties may serve as a stimulus for endogenous fructose production. To test this, we performed short-term experiments in ethanol-naive wild-type mice that were administered oral ethanol at varying doses (1–3 g kg^−1^) via gavage (Fig. 2b). Portal vein osmolality was measured 15 min after gavage. Ethanol significantly increased portal osmolality in a dose-dependent manner. Notably, when ethanol was administered in a more diluted form (that is, reduced from 25% to 5% concentration), the rise in osmolality was markedly attenuated, underscoring the concentration-dependent nature of ethanol’s osmotic effects.

Furthermore, twice-daily oral administration of undiluted ethanol over four consecutive days led to a significant increase in hepatic AR protein expression across all tested doses (1–3 g kg^−1^) (Fig. 2c). This effect was not observed when ethanol was diluted, implicating the hyperosmolarity of ethanol as a critical driver of AR induction and subsequent polyol pathway activation. Consistent with this mechanism, ethanol exposure resulted in a dose-dependent increase in hepatic sorbitol and fructose levels (Fig. 2d,e), supporting the hypothesis that ethanol promotes endogenous fructose production through osmotically mediated activation of the polyol pathway.

### Reduced ethanol preference and consumption in AR KO mice

To assess the contribution of endogenous fructose production to ethanol-related behaviours, we examined AR KO mice—both male and female—using the same behavioural assays applied to KHK-A/C KO mice. AR heterozygous animals, which express intermediate levels of AR relative to wild-type and AR KO mice (Fig. 2f), provided an opportunity to evaluate potential gene dosage effects.

Ethanol preference and intake were assessed using a two-bottle choice paradigm. Results revealed a dose-dependent relationship between AR expression and ethanol consumption, particularly at ethanol concentrations exceeding 6% (Fig. 2g–i for male mice; Fig. 2j–l for female mice). Although previous studies have established that pharmacological inhibition of AR confers protection against ALD, the role of AR in modulating ethanol preference and intake has not previously been evaluated in AR-deficient mice.

**Extended Data Fig. 5:**
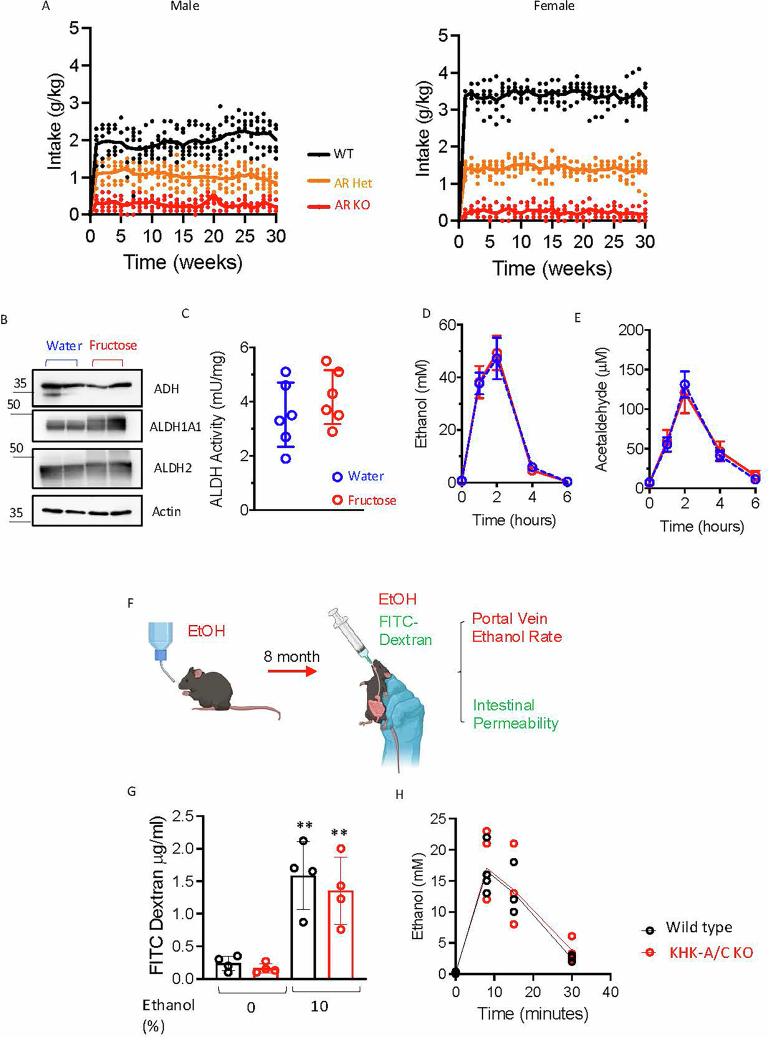
Ethanol intake, metabolism, and gut permeability in genetic and dietary AR and KHK-A/C KO models. **A**) 30-week ethanol intake in wild type (black), AR heterozygous (AR Het, orange) and AR knockout (AR KO, red) male and female mice. **B**) Representative western blot for liver ADH, ALDH1A1, ALDH2 and actin control in water and fructose (5.5 % w/v) fed mice for 60 weeks. **C**) Hepatic aldehyde dehydrogenase activity in water and fructose (5.5 % w/v) fed mice for 60 weeks. **D**) Time-response ethanol levels in plasma in water and fructose (5.5 % w/v) fed mice for 60 weeks and following an oral ethanol (3 g/kg) gavage. **E**) Time-response acetaldehyde levels in plasma in water and fructose (5.5 % w/v) fed mice for 60 weeks and following an oral ethanol (3 g/kg) gavage. **F**) Wild type (WT, black) and KHK-A/C KO (red) mice were on water control (0%) or exposed to ethanol for 8 months. After the conditioning period, mice were injected with either ethanol (3 g/kg) or FITC-Dextran and sedation determined by LORR and body temperature. **G**) Systemic FITC 2-hours after administration. **H**) Portal vein ethanol over a 30-minute period after oral gavage of ethanol. The data are shown as individual values (**A, G–H**) and as mean ± standard deviation (SD) (**C–E, G**). Panels **A** and **G** were analyzed by two-tailed t-test, and panels **D, E**, and **H** were analyzed by two-way ANOVA with Tukey’s post hoc multiple comparisons test. *P < 0.05, **P < 0.01; NS, not significant versus WT. Sample size: n = 6 mice per group (**A–E**), n = 4 mice per group and time point for FITC (**G**), and n = 3 mice per group and time point for EtOH (**H**). Panel **f** created with BioRender.com. Source data

Our data demonstrate that both daily and cumulative ethanol intake over a 30-week period were significantly reduced in AR KO mice compared with heterozygote and wild-type controls (Extended Data Fig. 5a). These findings support a critical role for polyol pathway activation, and, by extension, endogenous fructose production, in reinforcing ethanol consumption and suggest that AR activity contributes to ethanol-seeking behaviour.

### Role of liver fructose metabolism in regulating ethanol preference and intake

**Fig. 3:**
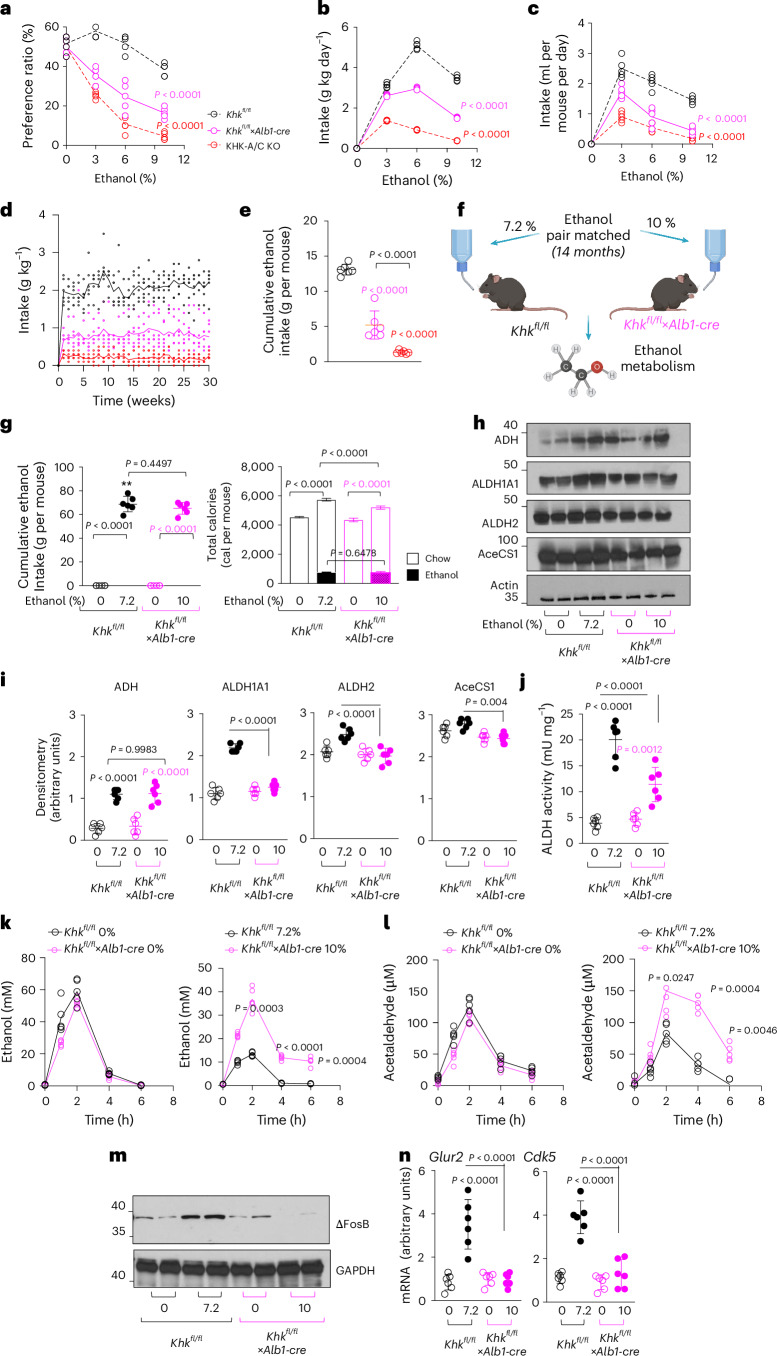
Hepatic determinants associated with endogenous fructose metabolism drive ethanol intake and preference and regulate ethanol metabolism. **a**, Ethanol-water two-bottle choice preference ratio of male wild-type (black), *Khk*^*fl/fl*^*×Alb1-cre* (pink) and KHK-A/C KO (red) mice at three different ethanol concentrations (vol/vol 3%, 6% and 10%). **b**, Average ethanol intake in g kg day^−1^ in the same groups as in **a**. **c**, Average ethanol intake in ml per mouse per day in the same groups as in **a**. **d**,**e**, Cumulative 30-week ethanol intake presented over time (**d**) and collapsed (**e**). **f**, Schematic design of ethanol exposure (one bottle, 14 months) of ethanol pair-matched *Khk*^*fl/fl*^ mice (7.2%, black) and *Khk*^*fl/fl*^×*Alb1-cre* (10%, pink) mice. **g**, Total (chow and ethanol-derived) caloric intake (one bottle, 14 months) by water control and ethanol pair-matched *Khk*^*fl/fl*^ mice (7.2%, black) and *Khk*^*fl/fl*^×*Alb1-cre* (10%, pink) mice. **h**,**i**, Representative western blot (**h**) and densitometry (**i**) for ADH, ALDH1A1, ALDH2, AceCS1 and actin loading control in the same mouse groups as in **f**. **j**, Hepatic ALDH activity in the same mouse groups as in **f**. **k**, Time-response ethanol levels in plasma in water control (0% ethanol, left) and ethanol pair-matched (right) *Khk*^*fl/fl*^ and *Khk*^*fl/fl*^×*Alb1-cre* mice following an oral ethanol (3 g kg^−1^) gavage. **l**, Time-response acetaldehyde levels in plasma in water control (0% ethanol, left) and ethanol pair-matched (right) *Khk*^*fl/fl*^ and *Khk*^*fl/fl*^×*Alb1-cre* mice following an oral ethanol (3 g kg^−1^) gavage. **m**, Representative western blot for ∆FosB and GAPDH loading control in the nucleus accumbens from 14-month water control and ethanol pair-matched *Khk*^*fl/fl*^ (7.2%) and *Khk*^*fl/fl*^×*Alb1-cre* (10%) mice. **n**, mRNA expression of ∆FosB target genes (*Glur2* and *Cdk5*) in the nucleus accumbens of 14-month water control and ethanol pair-matched *Khk*^*fl/fl*^ (black, 7.2%) and *Khk*^*fl/fl*^×*Alb1-cre* (pink, 10%) mice. The data are shown as individual values. Mean ± s.d. is additionally presented in **e**–**l**. Data were analysed in **a**–**g**, **i**, **j** and **n** using one-way ANOVA or two-way ANOVA (**g**) with Tukey’s multiple comparison post hoc test. Data in **k** and **l** were analysed using paired two-sided *t*-test between strains for each timepoint. Data were obtained from three separate studies and pooled together for statistical analysis. Sample size: *n* = 5–6 mice per group. See also Extended Data Fig. 8. Panels **a** and **f** created with Biorender.com. Source data

We and others have previously demonstrated that fructose metabolism in the gut and liver plays a critical role in regulating sugar intake and preference^10^. To delineate the tissue-specific contribution of this pathway to ethanol preference, we used mice with targeted deletion of KHK-A/C in either the liver or the intestine. Specifically, we compared global KHK-A/C KO mice with liver-specific KHK-A/C KO mice (*Khk*^*fl/fl*^×*Alb1-cre*) and their control littermates (*Khk*^*fl/fl*^) using a two-bottle choice test. As shown in Fig. 3a–c, *Khk*^*fl/fl*^×*Alb1-cre* mice exhibited significantly lower ethanol preference relative to controls, resulting in reduced ethanol intake over the 30-week study period (Fig. 3d,e). Although the reduction was less pronounced than that observed in systemic KHK-A/C KO mice, these findings underscore the importance of hepatic fructose metabolism in modulating ethanol preference.

To investigate the underlying mechanisms, we assessed ethanol metabolism in ethanol pair-matched *Khk*^*fl/fl*^ and *Khk*^*fl/fl*^×*Alb1-cre* mice. Mice received ethanol at concentrations of 10% vol/vol (*Khk*^*fl/fl*^*×Alb1-cre*) and 7.2% vol/vol (*Khk*^*fl/fl*^) to achieve equal total ethanol intake over 14 months (Fig. 5a). Both genotypes consumed ~75 g of ethanol per mouse, with matched caloric intake from ethanol and chow (Fig. 3f,g).

We then evaluated the expression of key enzymes involved in ethanol metabolism. Hepatic expression of alcohol dehydrogenase (ADH) (Fig. 3h,i) and cytochrome P450 2E1 (CYP2E1) (Extended Data Fig. 4) was similarly induced in both genotypes, indicating comparable conversion of ethanol to acetaldehyde. However, in the intestine, CYP2E1 expression remained significantly elevated in KHK-A/C KO mice compared with wild-type controls (Extended Data Fig. 4b), irrespective of ethanol exposure. Ethanol-treated wild-type mice exhibited a ~ 60% reduction in intestinal CYP2E1 expression compared with ethanol-naive controls, a compensatory response that was absent in KHK-A/C KO mice. This persistent increase in intestinal CYP2E1 may contribute to increased systemic acetaldehyde levels following ethanol exposure.

Further analysis revealed that ethanol-conditioned *Khk*^*fl/fl*^ mice exhibited robust upregulation of aldehyde dehydrogenase (ALDH) isoforms (ALDH1A1 and ALDH2), facilitating acetaldehyde detoxification. By contrast, expression of these enzymes was markedly reduced in ethanol-exposed *Khk*^*fl/fl*^*×Alb1-cre* mice (Fig. 3h–j), suggesting impaired acetaldehyde clearance in the absence of hepatic KHK-A/C. In addition, expression of acetyl-CoA synthetase 1 (AceCS1), responsible for converting acetate to acetyl-CoA, was modestly elevated in wild-type mice but remained unchanged in *Khk*^*fl/fl*^×*Alb1-cre* mice.

To evaluate the functional consequences of impaired ethanol metabolism, we measured circulating ethanol and acetaldehyde levels following an acute intraperitoneal ethanol injection (3 g kg^−1^). In ethanol-naive mice, no significant differences were observed between the genotypes (Fig. 3k,l, left). However, in ethanol-treated mice, *Khk*^*fl/fl*^*×Alb1-cre* mice exhibited significantly higher plasma ethanol and acetaldehyde levels compared with controls (Fig. 3k,l, right), consistent with reduced metabolic tolerance following chronic ethanol exposure. In addition, expression of ΔFosB and its downstream targets (*Glur2* and *Cdk5*) in the nucleus accumbens—a key marker of mesocorticolimbic reward pathway activation—was absent in ethanol-exposed *Khk*^*fl/fl*^×*Alb1-cre* mice (Fig. 3m,n). These findings imply a diminished central reward response to ethanol, at least in the nucleus accumbens, in the absence of hepatic KHK-A/C. Importantly, feeding fructose alone or administering acute ethanol injections did not alter ALDH expression or activity (Extended Data Fig. 5b–e), indicating that the effects of fructose metabolism on ethanol processing are contingent upon prior ethanol exposure.

### Role of intestinal fructose metabolism in regulating ethanol preference and intake

**Fig. 4:**
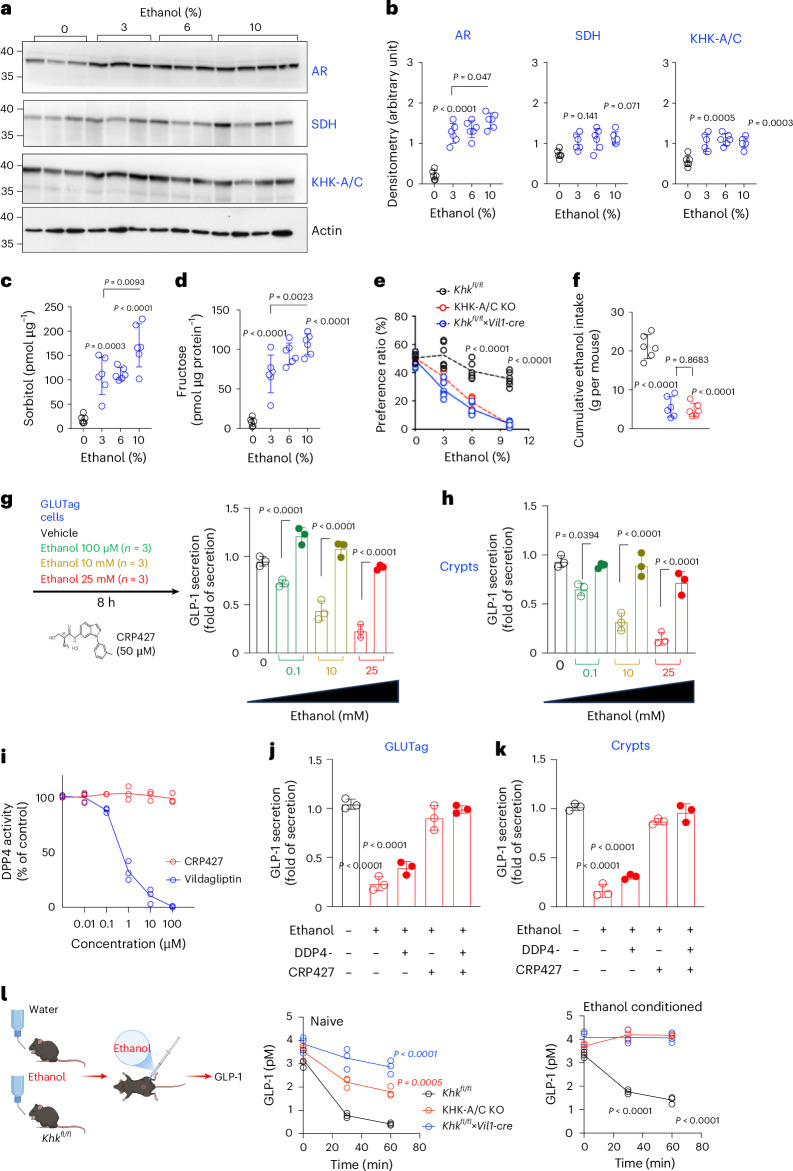
Intestinal determinants associated with endogenous fructose metabolism drive ethanol intake and preference. **a**,**b**, Representative western blot (**a**) and densitometry (**b**) for AR, SDH and KHK-A/C in the gut of wild-type mice consuming increasing concentrations of ethanol (3–10%). **c**,**d**, Intestinal sorbitol (**c**) and fructose (**d**) levels in wild-type mice consuming increasing concentrations of ethanol (3–10%). **e**, Ethanol-water two-bottle choice preference ratio of control *Khk*^*fl/fl*^ (black), whole body KHK-A/C KO (red) and gut-specific KHK-A/C KO (*Khk*^*fl/fl*^×*Vil1-cre*, blue) mice at three different ethanol concentrations (vol/vol 3%, 6% and 10%). **f**, Cumulative 30-week ethanol intake in the same mice as in **e**. **g**,**h**, GLP-1 release by GLUTag cells based on three independent replicates (**g**) and mouse intestinal crypts based on three independent replicates (**h**) exposed to increasing concentrations of ethanol in the presence of vehicle or the KHK-A/C inhibitor, CRP427. **i**, DPP-4 activity with the specific inhibitor vildagliptin (blue) or CRP427 (red). **j**,**k**, GLP-1 release by GLUTag cells based on three independent replicates (**j**) and mouse intestinal crypts (**k**) in the presence of vildagliptin (DDP4-) and/or CRP427 based on three independent replicates. **l**, Plasma GLP-1 in naive or ethanol treated *Khk*^*fl/fl*^ (black), KHK-A/C (red) and *Khk*^*fl/fl*^×*Vil1-cre* (blue) mice after an acute intraperitoneal injection of ethanol (3 g kg^−1^). The data are shown as individual values and were analysed using two-way ANOVA with Tukey’s post hoc multiple comparisons test. In **b**–**k**, values are presented as mean ± s.d. Data were obtained from three separate studies and pooled together for statistical analysis. Sample size: *n* = 3 (mice, L or plates, **g**–**k**) to 6 (mice, **b**–**f**) per group. See also Extended Data Fig. 5. Panel **l** created with BioRender.com. Source data

Similar to its effects in the liver, ethanol upregulates both AR and KHK-A/C expression in the jejunum in a dose-dependent manner (Fig. 4a,b). As expected, ethanol exposure significantly increases the levels of sorbitol and fructose in the jejunum (Fig. 4c,d). Consistent with the phenotype observed in liver-specific KO mice (*Khk*^*fl/fl*^*×Alb1-cre*), intestinal-specific deletion of KHK-A/C in *Khk*^*fl/fl*^×*Vil1-cre* led to a significant reduction in both ethanol intake and preference (Fig. 4e,f). These findings suggest that endogenous fructose metabolism in both the liver and intestine has a role in modulating ethanol consumption and preference in mice.

To explore the potential mechanisms underlying this intestinal effect, we investigated the role of the gut–brain hormonal axis. There is increasing evidence supporting the involvement of gut-derived glucagon-like peptide 1 (GLP-1) in reducing alcohol intake in both preclinical and clinical studies^25,26^. Using the murine L-cell line GLUTag and ex vivo intestinal preparations, we found that ethanol significantly suppressed basal GLP-1 secretion (Fig. 4g,h). Notably, this suppression was prevented by cotreatment with the KHK inhibitor CRP427, suggesting that KHK activity mediates ethanol-induced inhibition of GLP-1 release. Furthermore, the observed increase in GLP-1 secretion following KHK inhibition was independent of dipeptidyl peptidase-4 (DPP-4) activity, which degrades GLP-1 (Fig. 4i–k).

Importantly, the ethanol concentrations used in these in vitro studies (100 μM to 25 mM) fall within the physiological range observed in human plasma following alcohol consumption, underscoring the potential clinical relevance. In line with these findings, acute ethanol administration (3 g kg^−1^, intraperitoneal) significantly reduced circulating GLP-1 levels in both naive and ethanol-treated wild-type mice (Fig. 4l). This effect was notably blunted in both intestinal-specific and whole-body KHK-A/C KO mice. Collectively, these data suggest that ethanol suppresses GLP-1 secretion through a KHK-dependent mechanism in the intestine, and that inhibition of intestinal KHK restores GLP-1 signalling. This restoration may contribute to the reduced ethanol intake observed in KHK-A/C-deficient models.

### Effects of blocking liver KHK in ethanol metabolism and ALD

**Fig. 5:**
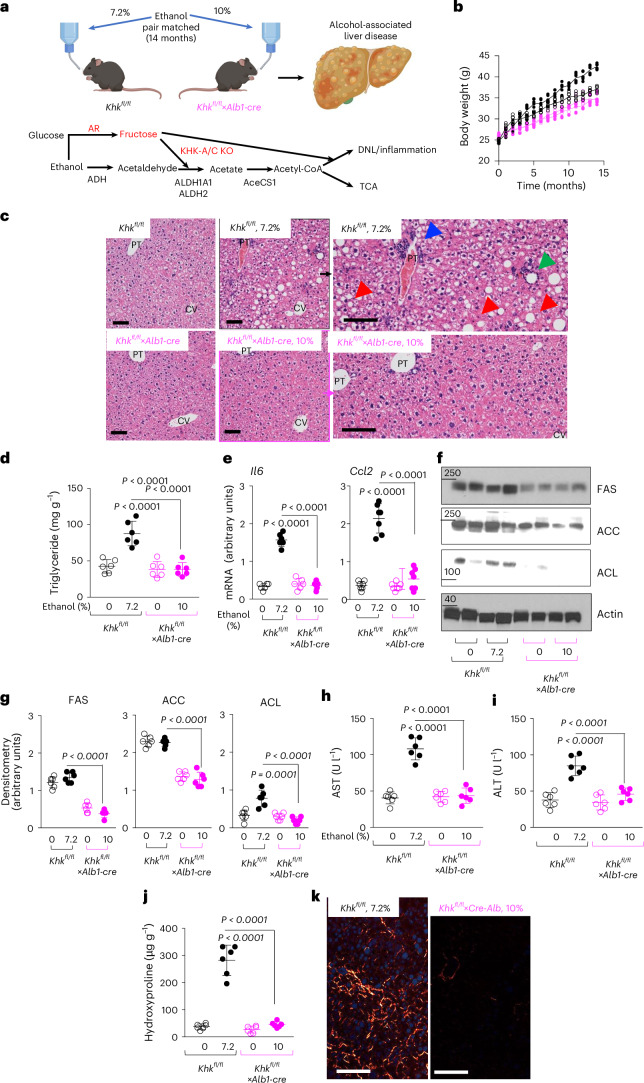
Blockade of hepatic fructose metabolism protects against ALD. **a**, Schematic with the study design and depiction of endogenous fructose production and metabolism in the liver via AR and the two main sites of regulation by fructose: ALDH expression or activity and promotion of de novo lipogenesis (DNL) and inflammation. **b**, Body weight gain in *Khk*^*fl/fl*^ mice (7.2%, black) and *Khk*^*fl/fl*^×*Alb1-cre* (10%, pink) on water or ethanol (pair-matched). **c**, Representative haematoxylin and eosin images in livers of mice from the same groups as in **b**. Right: Image magnification demonstrating steatotic (red arrows) areas, lipogranulomas (green arrow) and inflammation (blue arrows) PT: portal triad, CV: central vein. Scale bar: 20 µM. **d**, Liver triglyceride concentration in water control and 14-month ethanol pair-matched *Khk*^*fl/fl*^ mice (7.2%, black) and *Khk*^*fl/fl*^×*Alb1-cre* (10%, pink) mice. **e**, mRNA expression of pro-inflammatory cytokines (*Il6*) and chemokines (*Ccl2*) in mice from the same groups as in **b**. **f**,**g**, Representative western blot (**f**) and densitometry (**g**) for lipogenic enzymes FAS, ACC, ACL and actin loading control in the liver of mice from the same mouse groups as in **b**. **h**,**i**, Plasma AST (**h**) and ALT (**i**) levels in mice from the same groups as in **b**. **j**, Liver hydroxyproline levels in mice from the same groups as in **b**. **k**, Representative PSR image from 14-month ethanol-exposed *Khk*^*fl/fl*^ mice (7.2%) and *Khk*^*fl/fl*^×*Alb1-cre* (10%) mice. Scale bars, 20 µm. The data in **b**, **d** and **f**–**i** are shown as individual values and were analysed using one-way ANOVA with Tukey’s post hoc multiple comparisons test. Values in **b**–**d** and **f**–**i** are presented as mean ± s.d. **P < 0.01 versus respective water (0% ethanol) control; ##*P* < 0.01. Data were obtained from two separate studies and pooled together for statistical analysis. Sample size: *n* = 6 mice per group. See also Extended Data Figs. 6–7. Panel **a** created with BioRender.com. Source data

In addition to its established role in regulating hepatic ALDH expression, fructose has been shown—by our group and others—to have potent lipogenic and pro-inflammatory properties^10,15^. To investigate the contribution of hepatic fructose metabolism in ALD, we examined the effects of chronic ethanol exposure in mice with liver-specific deletion of KHK-A/C. Given that ALD is characterized by both hepatic steatosis and inflammation, we assessed liver injury and function after 14 months of single-bottle oral ethanol exposure in volume-matched groups (10% ethanol for *Khk*^*fl/fl*^*×Alb1-cre* and 7.2% ethanol for *Khk*^*fl/fl*^ mice) (Fig. 5a). Notably, no significant differences in body weight gain were observed between ethanol-consuming, pair-fed *Khk*^*fl/fl*^*×Alb1-cre* and *Khk*^*fl/fl*^ mice (Fig. 5b). In addition, assessment of intestinal permeability revealed no substantial differences between the genotypes, indicating that intestinal barrier function, and thus the contribution of the gut to systemic ethanol exposure, remained comparable across control and ethanol-treated wild-type and KHK-A/C-deficient mice over the 8-month study period (Extended Data Fig. 5f–h).

As shown in Fig. 5c–j, *Khk*^*fl/fl*^ mice developed pronounced steatosis, with macrovesicular lipid accumulation predominantly in hepatic zones 2 and 3, accompanied by inflammatory foci in zones 1 and 2, portal triad inflammation and the presence of lipogranulomas. These histological abnormalities were accompanied by significant biochemical changes, including elevated hepatic triglyceride content (Fig. 5d), increased expression of pro-inflammatory cytokines (*Il6*) and chemokines (*Ccl2*) and upregulation of key lipogenic enzymes such as FAS, ACC and ATP citrate lyase (Fig. 5e–g). Liver injury in *Khk*^*fl/fl*^ mice was further confirmed by increased transaminase levels (Fig. 5h,i) and markers of fibrosis, including increased hepatic hydroxyproline and enhanced picrosirius red (PSR) staining (Fig. 5j,k). By contrast, these pathological changes were largely absent in ethanol-treated *Khk*^*fl/fl*^*×Alb1-cre* mice, suggesting that hepatic KHK-A/C activity is required for ethanol-induced liver injury.

**Extended Data Fig. 6:**
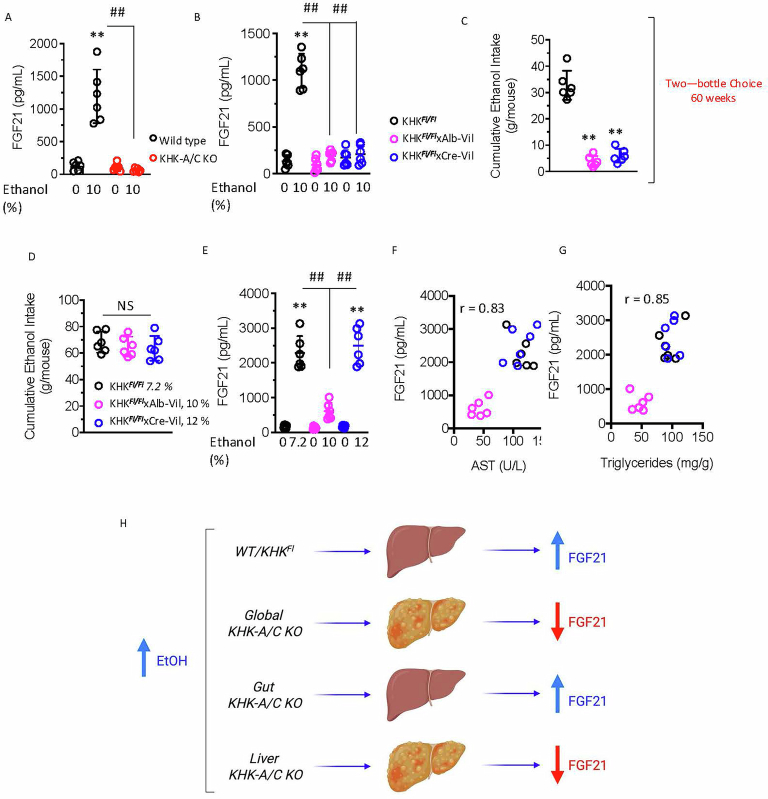
Reduced ethanol preference and intake in KHK-A/C KO mice is independent of FGF21. **A**) Plasma FGF21 levels in wild type and whole body KHK-A/C KO mice on ethanol/water two bottle choice preference study for 60 weeks. **B**) Plasma FGF21 levels in KHK^*Fl/Fl*^ (black), KHK^*Fl/Fl*^xCre-Alb (pink) and KHK^*Fl/Fl*^xCre-Vil (blue) mice on ethanol/water two bottle choice preference study for 60 weeks. **C**) Cumulative ethanol intake in KHK^*Fl/Fl*^ (black), KHK^*Fl/Fl*^xCre-Alb (pink) and KHK^*Fl/Fl*^xCre-Vil (blue) mice on ethanol/water two bottle choice preference study for 60 weeks. **D**) Cumulative ethanol intake in ethanol-paired KHK^*Fl/Fl*^ (7.2 %, black), KHK^*Fl/Fl*^xCre-Alb (10 %, pink) and KHK^*Fl/Fl*^xCre-Vil (12 %, blue) mice for 60 weeks. **E**) Plasma FGF21 levels in ethanol-paired KHK^*Fl/Fl*^ (7.2 %, black), KHK^*Fl/Fl*^xCre-Alb (10 %, pink) and KHK^*Fl/Fl*^xCre-Vil (12 %, blue) mice for 60 weeks. **F**) Linear correlation between plasma FGF21 and plasma AST in ethanol-paired KHK^*Fl/Fl*^ (7.2 %, black), KHK^*Fl/Fl*^xCre-Alb (10 %, pink) and KHK^*Fl/Fl*^xCre-Vil (12 %, blue) mice for 60 weeks. **G**) Linear correlation between plasma FGF21 and liver triglycerides in ethanol-paired KHK^*Fl/Fl*^ (7.2 %, black), KHK^*Fl/Fl*^xCre-Alb (10 %, pink) and KHK^*Fl/Fl*^xCre-Vil (12 %, blue) mice for 60 weeks. **H**) Proposed mechanism whereby hepatic KHK induces the production and release of ethanol-dependent FGF21. The data are presented as the means ± SEM and analyzed by two-tail t-test (**A**) or 2Way ANOVA (**B-G**) with Tukey post hoc multiple comparison analysis. **P < 0.01 versus WT or KHK^*Fl/Fl*^, ## *P* < 0.01, NS = not significant. *n* = 6 mice per group. Source data

**Extended Data Fig. 7:**
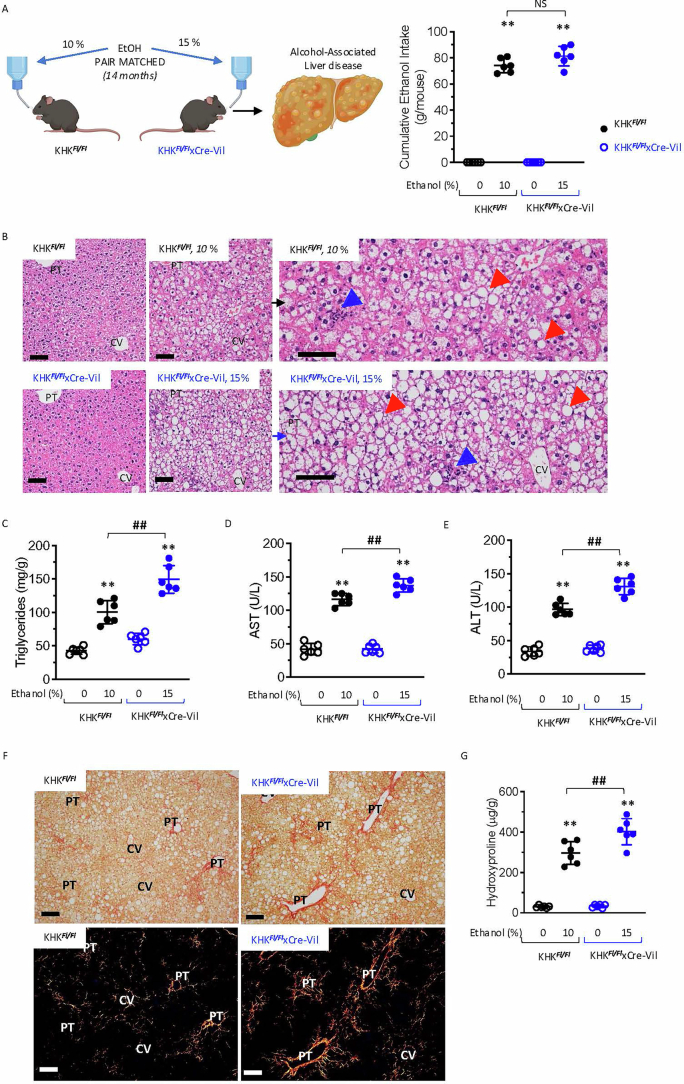
Effect of intestinal blockade of fructose metabolism in alcohol-associated liver disease. **A**) Study design and cumulative ethanol intake in ethanol pair matched KHK^*Fl/Fl*^ mice (10%, black) and KHK^*Fl/Fl*^xCre-Vil (15%, blue) **B**) Representative H&E images in livers of mice from the same groups as in **A**). Right side: Image magnification demonstrating steatotic (red arrows) areas and inflammation (blue arrows) PT: Portal triad, CV: Central Vein. Bar Size: 20 µM **C**) Liver triglyceride concentration in water control and 14-month ethanol pair-matched KHK^*Fl/Fl*^ mice (10%, black) and KHK^*Fl/Fl*^xCre-Vil (15%, blue) mice. D-E) Plasma AST and ALT levels in mice from the same groups as in **A**). **F**) Representative picro-sirius red (PSR) image from 14-month ethanol exposed KHK^*Fl/Fl*^ mice (10%) and KHK^*Fl/Fl*^xCre-Vil (15%) mice. **I**) Liver hydroxyproline levels in mice from the same groups as in **F**). Data are presented as individual values and analyzed by 2-Way ANOVA with Tukey post hoc multiple analysis.) **P < 0.01 versus respective water (0 % ethanol) control, ## *P* < 0.01. NS= not significant n = 6 mice per group. Panel **a** created with BioRender.com. Source data

Recent studies in both humans and rodents have identified FGF21, a hormone primarily secreted by the liver and adipose tissue, as a negative regulator of fructose and ethanol intake that also protects against liver disease^27^. The regulatory effect on intake is mediated via the β-Klotho–FGFR1c receptor complex in brain regions involved in reward and metabolism, such as the hypothalamus and nucleus accumbens^28^. To explore the role of FGF21 in ethanol consumption and ALD, we analysed FGF21 levels across different genetic models of fructose metabolism. Under voluntary two-bottle choice conditions, FGF21 levels were significantly higher in wild-type and *Khk*^*fl/fl*^ mice compared with whole-body KHK-A/C KO, *Khk*^*fl/fl*^×*Alb1-cre* and *Khk*^*fl/fl*^×*Vil1-cre* mice (Extended Data Fig. 6a–c). However, when ethanol intake was pair-matched (that is, absence of free access), *Khk*^*fl/fl*^×*Vil1-cre* mice exhibited increased FGF21 levels in response to ethanol (Extended Data Fig. 6d,e). Consistently, when *Khk*^*fl/fl*^×*Vil1-cre* mice were forced to consume ethanol chronically, they developed similar features of ALD as *Khk*^*fl/fl*^ mice (Extended Data Fig. 7a–g), suggesting a possible interactive effect with stress.

These findings demonstrate that liver-specific KHK deletion not only protects against ethanol-induced steatosis and inflammation but also prevents upregulation of hepatic and circulating FGF21. This is consistent with previous observations in fructose-fed KHK-A/C KO mice, where FGF21 induction was absent in both whole-body and hepatocyte-specific, but not intestine-specific, KHK-deficient models^10^. Together, the data suggest that the FGF21 response to ethanol (and fructose) is driven not by the presence of these substrates per se but by the resulting liver injury. Accordingly, in the absence of hepatic damage, as seen in *Khk*^*fl/fl*^×*Alb1-cre* mice, FGF21 is not induced. Supporting this, FGF21 levels were found to correlate positively with plasma AST and hepatic triglyceride content (Extended Data Fig. 6f–h), further reinforcing the link between liver injury and FGF21 activation.

### Targeted KHK-A/C deletion reverses ethanol preference and intake in mice

**Fig. 6:**
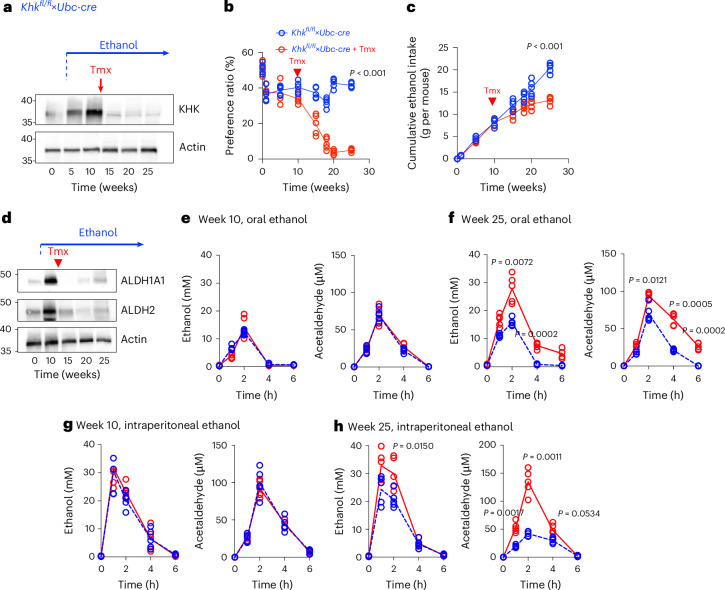
Targeted KHK-A/C deletion reverses ethanol preference and intake in mice. **a**, Representative western blot for liver KHK and actin loading control in *Khk*^*fl/fl*^×*Ubc-cre* mice before (0–10 weeks) and after (10–25 weeks) tamoxifen (Tmx) treatment. **b**, Weekly ethanol (10%)-water two-bottle choice preference ratio of *Khk*^*fl/fl*^×*Ubc-cre* control (blue) or Tmx-treated (red) mice. Arrow denotes time for Tmx treatment (week 10). **c**, Cumulative ethanol intake in the same mice as in **b**. Arrow denotes time for Tmx treatment (week 10). **d**, Representative western blot for liver ALDH1A1, ALDH2 and actin loading control in *Khk*^*fl/fl*^×*Ubc-cre* mice before (0–10 weeks) and after (10–25 weeks) Tmx treatment. **e**–**h**, Time-response ethanol (left) and acetaldehyde (right) levels in plasma in *Khk*^*fl/fl*^×*Ubc-cre* control (blue) or Tmx-treated (red) mice at week 10 (before Tmx treatment) following an oral (**e**,**f**) or intraperitoneal (**g**,**h**) ethanol (3 g kg^−1^) administration. Data in **b**, **c** and **e**–**h** are shown as individual values and were analysed using a paired two-sided *t*-test for each timepoint. In **b** and **c**, values are presented as mean ± s.d. Data were obtained from two separate studies and pooled together for statistical analysis. Sample size: *n* = 5 mice per group. Source data

The experiments in the above sections were conducted to determine whether the blockade of KHK could prevent mice from consuming ethanol. To test whether interventional approaches targeting KHK could be beneficial for reducing ethanol consumption in mice preconditioned to ethanol, we conducted a follow-up study in which the deletion of KHK expression was induced in ethanol-preferring mice. To that end, we used tamoxifen-inducible Cre recombinase-expressing *Khk*^*fl/fl*^ mice under the control of the ubiquitin promoter (*Khk*^*fl/fl*^×*Ubc-cre*). Hepatic KHK expression is eliminated in *Khk*^*fl/fl*^×*Ubc-cre* mice 10–14 days after tamoxifen treatment (Fig. 6a). Control *Khk*^*fl/fl*^ and tamoxifen-inducible *Khk*^*fl/fl*^×*Ubc-cre* mice showed no differences in ethanol intake and preference before tamoxifen treatment (Fig. 6b). However, after induction of KHK deletion, the preference for ethanol in *Khk*^*fl/fl*^×*Ubc-cre* mice and their accumulated ethanol intake were significantly reduced in a time-dependent manner (Fig. 6b,c). The deletion of KHK was accompanied by a marked downregulation in the expression of ALDH1A1 and ALDH2 (Fig. 6d), as well as reduced ethanol metabolism after acute ethanol administration (3 g kg^−1^) via oral gavage (Fig. 6e,f) or an acute intraperitoneal injection, bypassing any potential issues derived from compromised intestinal function after chronic ethanol exposure (Fig. 6g,h).

### Pharmacological inhibition of KHK-A/C reduces voluntary preference and ethanol intake in WT, cHAP mice and rats

**Fig. 7:**
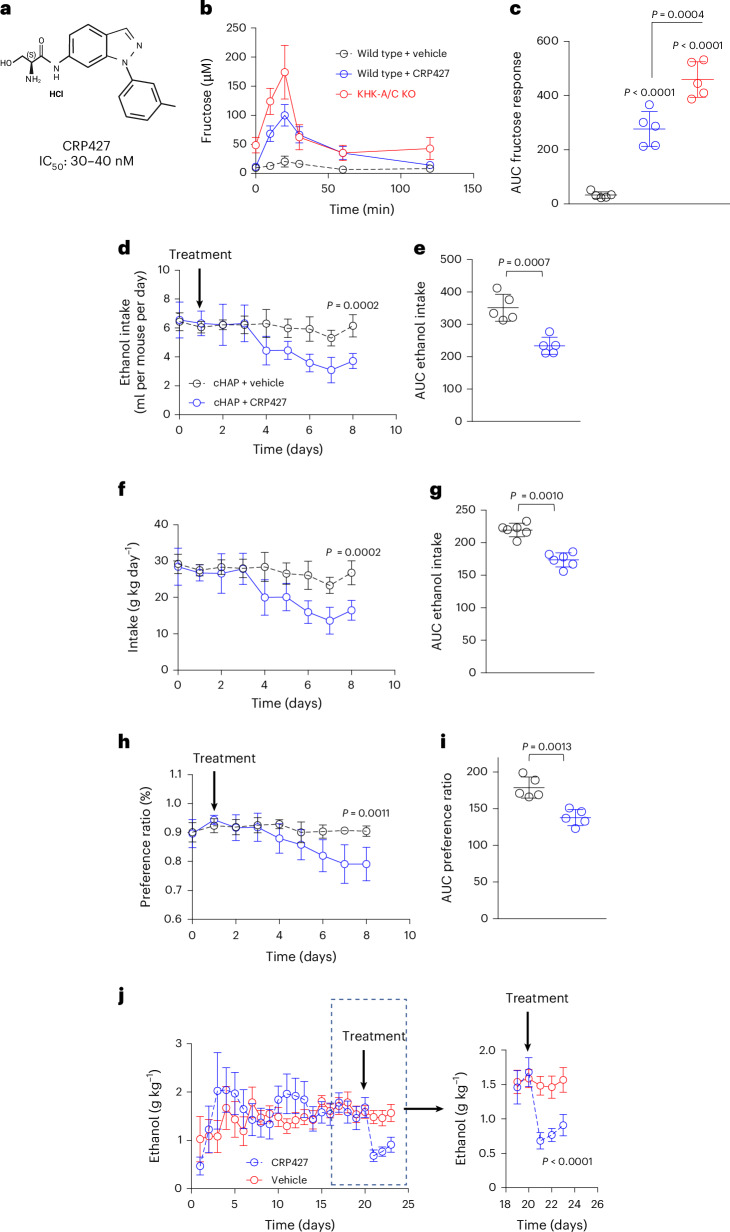
Pharmacological inhibition of KHK-A/C decreases intake and ethanol preference in cHAP mice and rats in an operant self-administration model. **a**, Chemical structure of KHK-A/C inhibitor CRP427. IC_50_, half-maximal inhibitory concentration. **b**,**c**, The 2-h systemic plasma fructose levels (**b**) and area under the curve (AUC) quantification (**c**) following a 1.5 g kg^−1^ oral fructose load in KHK-A/C KO (red) mice and wild-type mice receiving vehicle (black) or CRP427 (blue). **d**,**e**, Ethanol intake (ml per mouse per day) (**d**) and AUC quantification (**e**) in cHAP mice on an ethanol-water two-bottle choice preference study receiving vehicle (black) or CRP427 for eight consecutive days. **f**,**g**, Ethanol intake (g kg day^−1^) (**f**) and AUC quantification (**g**) in cHAP mice in an ethanol-water two-bottle choice preference study receiving vehicle (black) or CRP427 for eight consecutive days. **h**,**i**, Ethanol-water two-bottle choice preference ratio (**h**) and AUC quantification (**i**) in cHAP mice receiving vehicle (black) or CRP427 for eight consecutive days. **j**, Ethanol (g kg^−1^) consumed by rats in an operant self-administration model receiving vehicle (red) or CRP427 (blue) at day 20. The data in **b**–**j** are presented as mean ± s.d. and were analysed using one-way ANOVA with Tukey’s post hoc analysis (**c**) or a paired two-sided *t*-test for each timepoint (**d**–**j**). Sample size: *n* = 5 mice per group. See also Extended Data Fig. 8. Source data

In addition to the genetic approach of deleting KHK to test its effects on ethanol intake and preference in ethanol-conditioned mice, we aimed to determine whether pharmacological inhibition of KHK could also be relevant for reducing ethanol consumption. To this end, we tested the pharmacological blockade of KHK-A/C in a mouse model of high ethanol drinking using the crossed high alcohol preferring (cHAP) mouse model^29^. Pharmacological inhibition of KHK was achieved with CRP427, a compound generated by our group. CRP427 effectively inhibits KHK-C in the 30–40-nM range (Fig. 7a and Extended Data Table 1), is orally bioavailable and is taken up by the liver (Fig. 7b and Extended Data Table 1). Serum fructose levels following a fructose challenge (1.5 g kg^−1^) increased markedly in wild-type mice receiving CRP427, with plasma fructose ~40% lower than that observed in whole-body KHK-A/C KO mice (Fig. 7b,c). These results indicate that CRP427 inhibits fructose metabolism in vivo.

**Extended Data Fig. 8:**
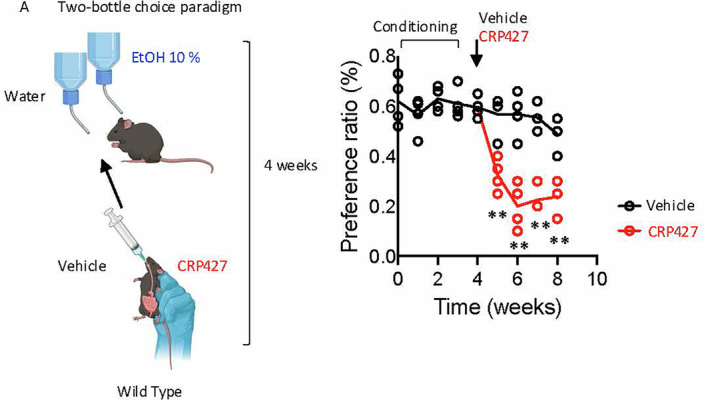
Effect of pharmacological inhibition of KHK on voluntary intake of ethanol in wild type mice. **A**) Study design: Ethanol preferring wild type (WT) mice were selected and conditioned to ethanol for 4-weeks. After the exposure period mice were subdivided into two groups with balanced ethanol intakes and subjected to either vehicle (black) or CRP427 (red). Right: Ethanol-water preference ratio over time before and after the administration of CRP427 or vehicle. Data are presented as individual values and analyzed by 2-Way ANOVA with Tukey post hoc multiple analysis.) **P < 0.01 versus vehicle *n* = 3 mice per group. Created with BioRender.com. Source data

The cHAP mouse model is characterized by free-choice drinking and moderate-to-high blood ethanol concentrations, similar to those observed in individuals with AUD^29^. We tested these ethanol-preferring cHAP mice in a two-bottle choice procedure (10% vol/vol ethanol versus water) for 2 weeks, selecting mice that showed a preference for ethanol of 90% or higher and intakes of nearly 30 g kg day^−1^, consistent with a prior report^29^. Mice were treated with either CRP427 or vehicle (water) for 5 days. As shown in Fig. 7d–j, CRP427 resulted in both a decrease in ethanol preference and overall ethanol intake compared with vehicle-treated cHAP mice. Notably, a similar result was observed when CRP427 was administered to wild-type mice that were previously conditioned to ethanol (Extended Data Fig. 8).

**Extended Data Fig. 9:**
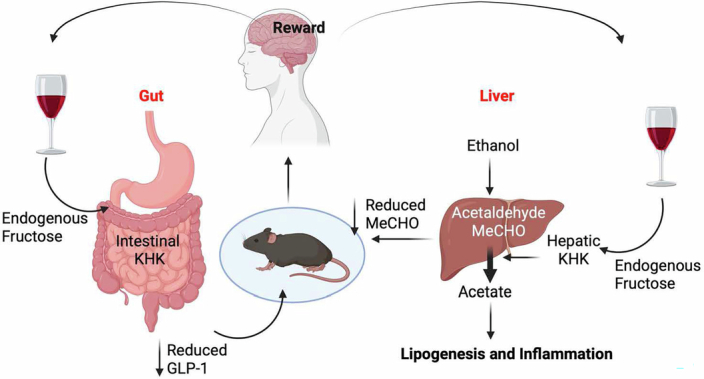
Schematic Showing a Role for Fructose Metabolism in Alcohol Use Disorder. Ethanol stimulates the endogenous production and metabolism of fructose in both the gut and liver. In the gut, fructose metabolism is essential for ethanol to suppress the release of GLP-1, a hormone known to reduce alcohol intake. In the liver, fructose metabolism promotes the expression of aldehyde dehydrogenases, enhancing ethanol clearance by lowering plasma acetaldehyde (MeCHO) levels, while also driving lipogenesis via acetyl-CoA derived from ethanol. When KHK-A/C is active, this dual effect—suppressed GLP-1 and reduced acetaldehyde—supports the activation of central reward pathways, increasing ethanol craving and intake. In contrast, inhibition of KHK-A/C leads to GLP-1 release from L-cells and accumulation of acetaldehyde. Together, these factors blunt the activation of the reward marker ∆FosB, and its down-stream targets, in the nucleus accumbens, diminishing the reinforcing effects of ethanol and reducing intake. Created with BioRender.com.

Furthermore, ethanol-preferring rats demonstrated a marked reduction in voluntary ethanol administration in an oral operant model of self-administration (Fig. 7j,k), suggesting a reduction in appetitive reinforcement. Collectively, our data demonstrate that genetic and pharmacological targeting of KHK could be potentially relevant in reducing ethanol intake and preference in models of excessive ethanol intake (Extended Data Fig. 9).

## Discussion

AUD is a major health problem worldwide. Interestingly, individuals who have a preference for alcohol also tend to have a preference for sugar^2,30^, and both substances can elicit reinforced responding (that is, they are reinforcing)^30^ as well as liver disease^11^. In this study, we investigated the possibility that these two substances are metabolically linked, and that alcohol may exert its effects, including reinforcement and reward, in part by driving fructose production and metabolism. A methodological difference between our study and others demonstrating synergistic effects on liver disease following combined ethanol and sugar administration^31^ is that we provide compelling data demonstrating an intertwined mechanism of action.

Our data indicate that alcohol preference is, at least in part, dependent on fructose metabolism. Both KHK-A/C KO mice and wild-type mice and rats treated with a KHK inhibitor showed reduced ethanol intake under free-choice conditions. These findings support a dual mechanism underlying the behavioural response to alcohol. First, blocking fructose metabolism in hepatocytes prevents the upregulation of ALDH, resulting in elevated acetaldehyde levels during heavy alcohol consumption. Inhibition of hepatic ALDH2 is associated with flushing reactions and decreased alcohol intake^32^. Conversely, acetaldehyde itself can be reinforcing^33^, and maintaining a critical level of acetaldehyde (that is, a reinforcing level of acetaldehyde^34^) may, in fact, be a limiting factor in ethanol intake. Notably, hepatocyte-specific KHK-A/C KO mice consumed more ethanol than systemic knockout mice, suggesting that fructose metabolism in non-hepatic tissues also has a role in regulating alcohol consumption. This effect seems to be associated with increased sensitivity to the ethanol sedative and hypothermic properties. Interestingly, the possibility that FGF21 may modulate the sedative or hypothermic effects of ethanol provides a compelling mechanism that could help explain our findings. FGF21 has been shown to activate the sympathetic nervous system, increasing energy expenditure and thermogenesis, as previously reported^35^. In a more directly relevant context, Choi et al.^36^ demonstrated that FGF21 counteracts alcohol intoxication by activating noradrenergic neurons, thereby promoting arousal and mitigating ethanol-induced sedation. These findings suggest that in models with impaired FGF21 signalling, such as KHK-A/C deficient mice, the lack of this counter-regulatory mechanism may enhance the sedative effects of ethanol, potentially contributing to reduced intake. Conversely, treatments that increase FGF21 signalling might not only reduce alcohol consumption but also blunt its sedative and thermoregulatory effects, offering a dual therapeutic benefit. Second, our data from intestinal KHK-A/C KO mice point to a gut–brain-axis mechanism, where ethanol-induced fructose metabolism impairs GLP-1 release from intestinal L-cells. GLP-1 is a key hormone known to suppress voluntary alcohol intake in both animal models and humans^25,26^. Our group has recently shown that the absence of fructose metabolism in the gut dramatically reduces the appetite of mice for fructose-containing sugars^10^ and, therefore, the absence of endogenous fructose metabolism in the gut may result in a greater aversion to both sugar and alcohol. We have also considered that fructose metabolism may exert direct effects within the brain, particularly in reward-associated regions such as the nucleus accumbens. In this regard, KHK-A/C is expressed in neurons^37^, and fructose can be endogenously produced in the brain through the polyol pathway^38^. Moreover, acetaldehyde can be metabolized from ethanol in the brain^39^ and, therefore, its clearance may depend on local fructose metabolism via KHK-A/C.

Another major finding was that the histopathology of ALD could be completely prevented in the hepatocyte-specific KHK-A/C KO mice. Currently, KHK inhibitors are being developed for the treatment of MASLD^40^, and our data suggest that they may also serve as a novel treatment for ALD. While others have reported similar findings^13,41^, we demonstrate that one key mechanism is that combining fructose with ethanol increases ethanol intake. Clinically, these findings suggest that individuals with AUD, especially those with liver disease, should consider limiting both alcohol and fructose intake. Notably, our study also found that the extra calories from ethanol and fructose reduced overall chow consumption. This decrease in food intake may model aspects of malnutrition seen in people with AUD, where deficiencies in key nutrients such as thiamine and folic acid may further worsen liver disease^42^.

We also found that ethanol can induce AR expression and stimulate fructose production, consistent with previous reports^18–20^. Notably, our previous work demonstrated that increasing portal vein osmolality through salt ingestion activates the polyol pathway, leading to hepatic fructose production and metabolic outcomes such as fatty liver and elevated blood pressure, effects that were absent in KHK KO mice despite identical salt intake^43^. These findings provide strong support for the concept that elevated portal vein osmolality serves as a physiological trigger for fructose generation and its downstream consequences. Under normal conditions, the liver does not express AR, but increased levels have been observed in individuals with ALD^18,19^, suggesting ethanol-driven AR induction. Previous studies have also shown that blocking AR can prevent ALD^19,20^. While this has been interpreted as implicating endogenous fructose in the development of ALD, the protective effects of AR inhibition may also involve other mechanisms, such as changes in redox potential owing to altered NAD^+^ and NADPH levels or reduced accumulation of sorbitol^44^. By using systemic and hepatocyte-specific KHK-A/C KO mice, we show that hepatic fructose metabolism has a key role in the development of ALD. Together with our findings on dietary fructose and MASLD, these data suggest that ALD and MASLD may share similar pathogenic pathways.

An interesting observation was that systemic KHK-A/C KO mice consumed very little ethanol, similar to their reduced intake of sugar, as previously reported^6,10^. By contrast, liver-specific KHK-A/C KO mice retained a strong preference for sugar despite being protected from MASLD^10^ and showed only a partial reduction in ethanol preference. One possible explanation involves the fructose-dependent upregulation of hepatic ALDH in response to ethanol. Blocking this upregulation, rather than inhibiting the enzyme directly, is unlikely to cause acetaldehyde accumulation except under conditions of excessive ethanol intake. This suggests that inhibiting liver KHK-A/C may reduce alcohol craving and heavy consumption while still permitting low-level intake without adverse effects. The significance of fructose metabolism in alcohol preference was further supported by experiments in selectively bred cHAP mice that consume >25 g kg day^−1^ of ethanol—levels known to yield blood ethanol concentrations >250 mg dl^−1^, comparable to those seen in individuals with AUD^29^. We found that treatment with the KHK-A/C inhibitor CRP427 significantly reduced voluntary alcohol consumption in these high-drinking animals, supporting our contention that this type of treatment might ameliorate drinking in people with an AUD as well. Notably, similar to humans, cHAP mice also show an elevated preference for sweet substances such as saccharin, although their sugar intake has not yet been quantified^45^.

A limitation of our study is that experiments were all conducted in laboratory mice and rats as opposed to in humans. However, prior studies have provided evidence that AR and hepatic fructose levels are high in humans with ALD^46^, and there is also evidence that blocking KHK may have benefit in reducing liver fat in individuals with metabolic dysfunction-associated steatohepatitis^40^. Thus, the similarities in physiology and behavioural responses observed between our studies and clinical literature suggest that these findings are relevant to humans as well. In addition, our findings support a role for GLP-1 as a mediator in the effects observed with KHK deficiency, consistent with emerging literature. However, we acknowledge that these results are correlative, and a definitive test of GLP-1 receptor involvement would require a targeted loss-of-function approach (for example, GLP-1R-floxed mice crossed with the KHK-A/C KO strain). Another limitation of our study is that the current work is primarily descriptive with respect to the neurobiological findings. Our goal was to establish a link between fructose metabolism, KHK activity and ethanol-related behaviours. While our results provide a strong foundation, further mechanistic studies are required to conclusively demonstrate a gut- or liver-derived satiety signal and to dissect the neural circuitry and molecular pathways underlying these effects.

In summary, we present evidence that ALD and alcohol intake and preference share dependence, at least in part, on fructose metabolism. Thus, sugar and alcohol intake and their metabolism appear to be intricately linked, indicating that blocking fructose metabolism may provide a novel approach for treating AUDs.

## Methods

### Study approval

All animal experiments were conducted with adherence to the National Institutes of Health Guide for the Care and Use of Laboratory Animals^47^. The animal protocol was approved by the Institutional Animal Care and Use Committee of the University of Colorado.

### Animals

AR (*Akr1b3*) KO mice were generated using CRISPR–Cas9 with the assistance of the Transgenic Core at the Gates Center for Regenerative Medicine, University of Colorado School of Medicine, and have been previously characterized^48^. KHK-A/C KO (B6;129-Khk^tm2Dtb^) and KHK-A KO (B6;129-Khk^tm2.1Dtb^) mice were originally developed by D. Bonthron^49^ and were bred and maintained at the University of Colorado with pure C57BL/6 for over seven generations to ensure the mice were on the B6 genetic background. Mice with *loxP* sequences flanking exons 3 and 4 of the *Khk* gene (*Khk*^*fl/fl*^) were generated as detailed previously^10^ by the Genomic Core at the University of Colorado Cancer Center. cHAP mice were selectively bred from a cross of the HAP1xHAP2 replicate lines provided by N. J. Grahame. These mice demonstrate blood ethanol concentrations during free-choice drinking that are reminiscent of those observed in ethanol-dependent humans^29^. All experimental mice were maintained in temperature- and humidity-controlled specific pathogen-free condition on a 14-h dark/10-h light cycle and at 25 °C, and allowed ad libitum access to normal laboratory chow (Harlan Teklad, 2920X). Water and food consumption was monitored daily and body weight recorded weekly for 30 weeks or 14 months depending on the study. All experiments were conducted with adherence to the National Institutes of Health Guide for the Care and Use of Laboratory Animals. In all studies, 7–10-week-old male mice (*n* = 5–6) were used. Food consumption was monitored daily and body weight was recorded. All animals in the study were phenotypically normal and generally healthy during the length of the study.

Two long-term studies were conducted: a two-bottle choice preference study for 30 weeks and a single-bottle ethanol intake study for 14 months.

For the two-bottle ethanol preference studies, mice were housed individually and provided with two similar 300-ml water bottles with ball-bearing sipper tubes filled with water for acclimation over a 5-day period. For the experiment, water in one of the bottles was substituted with water containing ethanol alone or in combination with sucralose (0.04% wt/vol) or fructose (5.5% wt/vol). Ethanol concentrations were subsequently increased as follows: 3% for 3 days, 6% for 5 days and 10% for up to 30 weeks. Every day after oral exposure, the position of the two bottles (regular and ethanol-containing water) was switched to control for side preference. The preference ratio was calculated as the ratio of volume of tastant consumed to the total volume consumed, that is, a score of 0.5 or 50% shows no preference.

For the 14-month study, mice were housed in pairs to account for socialization behaviours and exposed to a single bottle—no choice access—in which ethanol was gradually introduced as explained for the two-bottle choice preference studies. To compensate for differences in ethanol intake between strains, groups were pair-matched for ethanol intake by providing different concentrations of ethanol (10% in *Khk*^*fl/fl*^×*Alb1-Ubc* mice, 12% in *Khk*^*fl/fl*^×*Vil1-Ubc* mice and 7.2 % in *Khk*^*fl/fl*^ mice). This way, cumulative ethanol intake did not substantially differ between strains (65.1 ± 4.9 g versus 68.7 ± 6.4 g ethanol, respectively, *P* = NS).

Several short-term studies were conducted:

1. Assessment of portal vein osmolality: For portal vein studies, animals with the same weight were gavaged with water or varying concentrations of ethanol. Portal vein blood was collected 7 min after gavage from mice under isoflurane anaesthesia, and serum was obtained after centrifugation at 13,114*g* for 2 min at room temperature. Osmolality was determined with a freezing point osmometer (model 3300, Advanced Instruments).
2. CPP: The place preference apparatus (58 cm × 25 cm × 25 cm (length, width and height)) consists of three distinct rooms: two conditioning environments and a neutral area. Each conditioning environment measures 23 cm × 25 cm × 25 cm. The environments differed from each other in visual and tactile cues. One conditioning environment had white walls and a bar floor, while the other conditioning environment had black walls and a grid floor. The neutral area measured 12 cm × 25 cm × 25 cm and was grey with a dark grey Plexiglas floor. Each apparatus was in a sound-attenuated chamber under dim light conditions (20–25 lux) with masking noise. Data were collected using Med Associates software. Mice were handled for 2 min per day for 3 days before conditioning. Then, mice were pre-exposed to the CPP apparatus by allowing free access to the entire apparatus for 10 min on day 1, and the time spent in each room was recorded. Next, ten conditioning sessions were conducted; one 5 min session per day for 10 days on days 2–11, alternating between ethanol paired with one environment and saline paired with the other environment. The ethanol-paired side was the preferred side for half of the mice, and the non-preferred side for the other half; half of the mice received ethanol on the first day and half received saline on the first day. Mice were injected with either ethanol (2 g kg^−1^; 12.5 ml kg^−1^ of a 20% vol/vol solution of ethanol in saline) or equivolume of intraperitoneal saline and placed within the appropriate environment within 3 min. On day 12, mice were placed in the apparatus in an ethanol-free state and allowed free access to the entire apparatus for 10 min (1,200 s), and the time spent in each room was again recorded. Preference was calculated as the total time (s) spent in the ethanol-paired environment and the time (s) spent in the ethanol-paired environment after the test minus before the test (preference shift).
3. Analysis of locomotor activity: The locomotor apparatus consisted of an arena that measured 53 cm × 35 cm × 35 cm (length, width and height) within a sound-attenuated chamber under dim light conditions (20–25 lux). The same mice were used for the locomotor study, which followed 1 week after the CPP experiment. Mice were randomized for testing over 2 days. On the first day, they were placed in the apparatus in a drug-free state for 2 h, and, on the second day, mice were placed in the arena immediately after a single injection (2 g kg^−1^ intraperitoneally, as above) of ethanol. Locomotor activity (cm) was recorded in 10-min bins using Med Associates software.
4. Oral operant-ethanol self-administration study: Rats were first exposed to alcohol 10% in a two-bottle set up for 2 weeks to identify rats that liked alcohol. Thereafter, the ethanol-liking group was separately studied, in which they could self-administer a bolus of alcohol 15% by pressing a lever over a 90-min period in the dark. The number of presses and total volumes consumed were recorded. This was done five times per week. Rats that successfully learned the operant response were randomly divided into drug (*n* = 10) or vehicle (*n* = 9) group. CRP587 was given intraperitoneally at 5 mg kg^−1^ 90 min before the test session, while control rats received a vehicle. During the test session, the number of lever presses and volume of alcohol administered were determined over the next 90 min.
5. Assessment of ethanol-driven sedation: Ethanol-driven sedation was assessed using the LORR assay, which measures sensitivity to the sedative effects of ethanol. Mice were administered ethanol (3.6 g kg^−1^, intraperitoneally), and, once they became ataxic, they were gently placed in a supine position within V-shaped plastic troughs. Sleep time was defined as the duration from placement in the supine position until the mice were able to right themselves three times within a 30-s period, indicating recovery of the righting reflex.
6. Assessment of ethanol-induced hypothermia. Hypothermia was assessed in temperature-controlled chambers. Baseline body temperatures were recorded using a rectal probe, which was gently inserted for 5 s. Immediately following baseline measurement, the mice received an IP injection of ethanol (3.6 g kg^−1^) and were returned to the chambers. Rectal temperatures were subsequently monitored every 30 min for 5 h after the injection to assess changes in core body temperature over time.
7. Administration of the KHK inhibitor CRP427 to cHAP and wild-type mice: Animals were placed on a two-bottle choice paradigm with access to 10% ethanol and water. Ethanol and water intake were recorded daily during a 3-day baseline period for cHAP mice and over a 4-week period for wild-type mice. In cHAP mice (Fig. 7d–i), animals were orally gavaged with 150 µl of either vehicle (water) or CRP427 (50 mg kg^−1^) once daily for five consecutive days, starting on day 3 of the paradigm. Ethanol intake and preference were measured throughout the treatment period. In wild-type mice (Extended Data Fig. 8), a subset of animals (*n* = 6) was selected from an initial pool of 32 on the basis of a high ethanol preference (>65%) during the two-bottle choice test. After 5 weeks of ethanol access, the selected mice were divided into two groups (*n* = 3 each), with ethanol intake balanced across groups. Mice were then orally gavaged with 150 µl of either vehicle or CRP427 (50 mg kg^−1^) three times per week for 4 weeks. Ethanol preference was continuously monitored throughout the treatment period using the two-bottle choice protocol.
8. Analysis of intestinal permeability. Intestinal permeability was assessed using fluorescein isothiocyanate (FITC)-dextran. Mice were first exposed to ethanol for 8 months. To evaluate ethanol absorption, one cohort received a single oral dose of ethanol (4 g kg^−1^), and portal vein blood was collected 15–30 min later corresponding to the peak osmolality timepoints in our study. A second cohort (*n* = 4) was orally administered FITC-dextran (600 mg kg^−1^) to directly assess intestinal barrier function. A total of 2 h after administration, mice were anaesthetized with isoflurane, and systemic blood was collected via cardiac puncture. Plasma from PBS-dosed control mice was used to generate a standard curve. FITC-dextran concentrations in plasma were measured in duplicate using a spectrophotometer.

### Cell lines and primary cultures

Intestinal crypts from mice were obtained after dissecting the small intestine and washing it with cold PBS. Tissue was incubated in 2–10 mM EDTA in PBS for 20–30 min at 4 °C to dissociate the crypts that were then gently shaken in PBS to be released. The supernatant was filtered through a 40-µm cell strainer and centrifuged to collect the crypts. Crypts were resuspended in Matrigel and droplets (25–50 µl) plated in prewarmed culture plates in complete culture medium (advanced DMEM/F12 with GlutaMAX, HEPES, penicillin–streptomycin and supplements such as epidermal growth factor, Noggin and R-spondin). Crypts were cultured at 37 °C and, when organoids were formed, they were treated with ethanol at the doses indicated, and baseline and stimulated GLP-1 in the supernatant were measured using enzyme-linked immunosorbent assay (81508, Crystal Chem). GLUTAag cells were obtained from Millipore (SCC652) and maintained following the manufacturer’s protocol.

### Method details

#### Biochemical analysis

Blood was collected in microtainer tubes (BD) from cardiac puncture of mice under isoflurane, and serum was obtained after centrifugation at 13,000*g* for 2 min at room temperature. Serum parameters were performed biochemically following the manufacturer’s instruction (FGF21: R&D, MF2100, AST: Bioassay Systems, EASTR-100, ALT: Bioassay Systems, EALT-100, GLP-1 (81508, Crystal Chem)). Determination of parameters in tissue was performed in freeze-clamped tissues and measured biochemically following the manufacturer’s protocol (triglycerides (liver): Bioassay Systems, ETGA-200; uric acid: Bioassay Systems DIUA-250, sorbitol: Bioassay Systems ESBT-100 determined from deproteinized samples).

#### Histopathology

Formalin-fixed paraffin-embedded liver sections were stained with haematoxylin and eosin. Histological examination was performed through an entire cross section of liver from each mouse. Images were captured on an Olympus BX51 microscope equipped with a Macrofire digital camera (Optronics) using the PictureFrame Application 2.3 (Optronics). Composite images were assembled with the use of Adobe Photoshop. All images in each composite were handled identically.

#### Western blot

Protein lysates were prepared from mouse tissue using MAP kinase lysis buffer as previously described^50^. Protein content was determined by the BCA protein assay (Pierce). Total protein (50 μg) was separated by SDS–PAGE (10% wt/vol) and transferred to PVDF membranes (Bio-Rad). Membranes were first blocked for 1 h at 25 °C in 4% (wt/vol) instant milk dissolved in 0.1 % Tween-20 Tris-buffered saline, incubated with primary rabbit or mouse-raised antibodies (1:1,000 dilution in Tween-20 Tris-buffered saline) KHK (Sigma HPA007040; RRID:AB_1079185), ADH1 (Cell Signaling 5295; RRID: RRID:AB_10626624), ALDH1A1 (Cell Signaling 12035; RRID: RRID:AB_2797805), ALDH2 (Cell Signaling 18818; RRID:AB_2798804), AceCS1 (Cell Signaling 3658; RRID: RRID:AB_2222710), FAS (Cell Signaling 4233; RRID:AB_2100359), ACC (Cell Signaling 3676; RRID:AB_2219397), ACL (Cell Signaling 4332; RRID:AB_2223744), ∆FosB (Cell Signaling; 14695; RRID:AB_2798577) and actin (Cell Signaling 4968; RRID:2313904) and visualized using an anti-rabbit (7074; RRID:AB_2099233) or anti-mouse immunoglobulin G (IgG) (7076; RRID:AB_330924) horseradish-peroxidase conjugated secondary antibody (1:2,000, Cell Signaling) using the HRP Immunstar detection kit (Bio-Rad). Chemiluminescence was recorded with an Image Station 440CF and results analysed with the 1D Image Software (Kodak Digital Science).

#### Identification of CRP427 as a candidate drug to target KHK-A/C

Inhibition was measured by a coupled enzyme assay containing 33 mM triethanolamine, pH 7.4, 100 mM KCl, 6 mM MgCl_2_, 1.33 mM phosphoenolpyruvate, 0.1 mM ATP, 1.0 U ml^−1^ pyruvate kinase, 1.0 U ml^−1^ lactate dehydrogenase, 300 µM NADH and human KHK-C (100–300 nM; 0.05–0.15 U ml^−1^). Fructose at a final concentration of 1 mM was used to initiate 0.18-ml reactions in 96-well plates, and the change in absorbance at 340 nM was measured in a SpectraMax M5 plate reader. The activity was measured in the presence and absence of the inhibitor across a series of seven tenfold serial dilutions (0.0001–100 µM). The percentage activity was calculated by subtracting the rates with no substrate and dividing by the rates without inhibitor (solvent only DMSO (5% vol/vol)). The IC_50_ values were determined by plotting the percentage activity versus the log of inhibitor concentration with isotherms fit using a three-parameter nonlinear regression dose–response procedure.

#### Determination of CRP427 organ distribution

CRP427 and internal standard CRP511 were analysed via an Applied Biosystems Sciex 4000 (Applied Biosystems) equipped with a Shimadzu HPLC (Shimadzu Scientific Instruments, Inc.) and auto-sampler (Nexera X2 Sil-30AC). Organs were collected (liver, kidney and brain) and homogenates prepared by taking tissue weight and adding 2 volumes of PBS, such as 100 mg tissue and 200 μl PBS. The tissues were homogenized on ice using a handheld homogenizer (VWR 200). Homogenates (100 μl) were mixed with 200 μl extraction solution, methanol:acetonitrile (1:1) containing CRP511 as an internal standard, capped, vortex mixed (5 s) and centrifuged at 10,000*g* for 5 min (Eppendorf Centrifuge 5418), and the supernatant was analysed using liquid chromatography–tandem mass spectrometry. An extend-C18 column (4.6 × 50 mm) and a column guard were used; the column temperature was set to 40 °C. Solvent A was HPLC water with 10 mM NH_4_OAc and 0.1% formic acid; and solvent B was methanol:acetonitrile (1:1). The flowrate was set to 0.4 ml min^−1^. The chromatography conditions were 5% B for 0.5 min, ramped to 95% B at 6 min and held for 3.5 min at 95% B, then at 10.5 min return to 5% B and at 12 min stop. Mass spectrometry conditions included CRP427 eluting at 5.2 min with a transition of 311.2 → 224.0 *m*/*z* (declustering potential (DP) = 81, collision energy (CE) = 37, collision cell exit potential (CXP) = 14), and the internal standard CRP511 eluting at 5.6 min with a transition of 321.17 → 292.2 *m*/*z* (DP = 86, CE = 23, CXP = 20). Additional parameters included ion source gas 1 (GS1) = 35, ion source gas 2 (GS2) = 50, source temperature = 450 °C and collision-activated dissociation (CAD) = 12.

### Quantification and statistical analysis

#### Statistical analysis

All numerical data are presented as individual values or the mean ± standard error of the mean. Independent replicates for each data point (*n*) are identified in the figure legends. Data graphics and statistical analyses were performed using Prism 10, version 10.3.1 (GraphPad). Data without indications were analysed by one-way ANOVA with Tukey’s post hoc test. A value of *P* < 0.05 was regarded as statistically significant. Animals were randomly allocated in each group using randomizer (www.randomizer.org). Power calculations for the number of animals assigned to each group were based on our previous publications and designed to observe a >15% difference in the primary endpoint of each study between groups. In general, an *n* of 6 mice per group was used. Data distribution was assumed to be normal, but this was not formally tested. No animals were excluded from the study, and, whenever possible, experiments were performed in a blinded fashion. For example, for data analysis, except for western blot, single samples (plasma and homogenates) were first codified and decoded after analyses. Similarly, histological records and scoring were performed in a blinded fashion.

### Reporting summary

Further information on research design is available in the Nature Portfolio Reporting Summary linked to this article.

## Supplementary information

Reporting Summary

## Source data

Source Data Figs. 1–7 and Extended Data Figs. 1–8 Combined Statistical source data for Figs. 1–7 and Extended Data Figs. 1–8.

Unprocessed Blots Figs. and Extended Data Figs. Combined Unprocessed western blots with size markers.

## Figures and Tables

**Extended Data Table 1:**
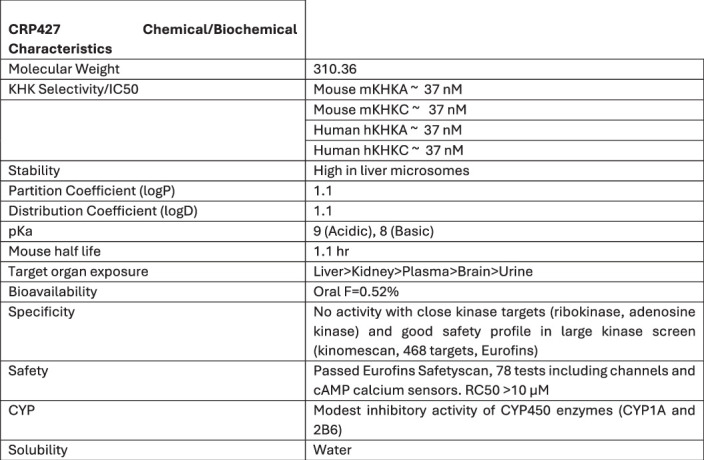
Chemical/Biochemical Characteristics of KHK-A/C inhibitor CRP427

## Data Availability

Further information and requests for resources and reagents should be directed and will be fulfilled by the corresponding author. Mouse lines generated in this study are available to any researcher upon reasonable request. Source data are provided with this paper. These data are also included in the Supplementary Information and are available upon request.

## Code availability

This study did not generate unique datasets or code.
