# Supplementary material for: Identification of a common ketohexokinase-dependent link driving alcohol intake and alcohol-associated liver disease in mice
Source: Nat Metab. 2025 Nov 10;7(11):2250–67. doi: 10.1038/s42255-025-01402-x (PMC12638252; doi:10.1038/s42255-025-01402-x)
Supplement: Supplementary file 1 — Reporting Summary [file 42255_2025_1402_MOESM1_ESM.pdf]

nature portfolio

Corresponding author(s): Miguel A. Lanaspa

Last updated by author(s): 07/07/2025

Reporting Summary

Nature Portfolio wishes to improve the reproducibility of the work that we publish. This form provides structure for consistency and transparency in reporting. For further information on Nature Portfolio policies, see our Editorial Policies and the Editorial Policy Checklist.

Statistics

For all statistical analyses, confirm that the following items are present in the figure legend, table legend, main text, or Methods section.

n/a	Confirmed	
☐	☒	The exact sample size (*n*) for each experimental group/condition, given as a discrete number and unit of measurement
☐	☒	A statement on whether measurements were taken from distinct samples or whether the same sample was measured repeatedly
☐	☒	The statistical test(s) used AND whether they are one- or two-sided<br>*Only common tests should be described solely by name; describe more complex techniques in the Methods section.*
☐	☒	A description of all covariates tested
☐	☒	A description of any assumptions or corrections, such as tests of normality and adjustment for multiple comparisons
☐	☒	A full description of the statistical parameters including central tendency (e.g. means) or other basic estimates (e.g. regression coefficient) AND variation (e.g. standard deviation) or associated estimates of uncertainty (e.g. confidence intervals)
☐	☒	For null hypothesis testing, the test statistic (e.g. F, t, r) with confidence intervals, effect sizes, degrees of freedom and P value noted<br>*Give P values as exact values whenever suitable.*
☒	☐	For Bayesian analysis, information on the choice of priors and Markov chain Monte Carlo settings
☒	☐	For hierarchical and complex designs, identification of the appropriate level for tests and full reporting of outcomes
☒	☐	Estimates of effect sizes (e.g. Cohen's d, Pearson's r), indicating how they were calculated

Our web collection on statistics for biologists contains articles on many of the points above.

Software and code

Policy information about availability of computer code

Data collection	No software was used
Data analysis	Data graphics and statistical analysis were performed using Prism 10 version 10.3.1 (GraphPad). Data without indications were analyzed by one-way ANOVA, Tukey post hoc test. A value of P < 0.05 was regarded as statistically significant. Animals were randomly allocated in each group using randomizer (www.randomizer.org). Power calculations for the number of animals assigned to each group were based on our previous publications and designed to observe a greater than 15% difference between groups. In general, an n of 6 mice per group was used. No animals were excluded from the study and whenever possible experiments were done in a blind fashion.

For manuscripts utilizing custom algorithms or software that are central to the research but not yet described in published literature, software must be made available to editors and reviewers. We strongly encourage code deposition in a community repository (e.g. GitHub). See the Nature Portfolio guidelines for submitting code & software for further information.

Data

Policy information about availability of data

All manuscripts must include a data availability statement. This statement should provide the following information, where applicable:

- Accession codes, unique identifiers, or web links for publicly available datasets
- A description of any restrictions on data availability
- For clinical datasets or third party data, please ensure that the statement adheres to our policy

Lead Contact:
Further information and requests for resources, reagents, or data should be directed to and will be fulfilled by the corresponding author, Dr. Miguel A. Lanaspa (Miguel.lanaspagarcia@cuanschutz.edu).

Materials Availability:
All mouse lines generated in this study are available to the scientific community upon reasonable request, in accordance with institutional guidelines and material transfer agreements (MTAs) where applicable. We are committed to supporting reproducibility and collaboration in research and will make these resources accessible to qualified investigators.

Data Availability:
All data reported in this paper will be made available by the corresponding author upon request. This includes raw and processed datasets used in figures, tables, and supplementary materials. We welcome inquiries for additional data that may assist in reanalysis or integration with other studies.

Code Availability:
This paper does not report original code. However, any additional information necessary to reanalyze or interpret the data presented in this study is available from the corresponding author upon request.

We are committed to transparency, openness, and collaborative science, and will make every reasonable effort to respond promptly and thoroughly to all requests.

Research involving human participants, their data, or biological material

Policy information about studies with human participants or human data. See also policy information about sex, gender (identity/presentation), and sexual orientation and race, ethnicity and racism.

Reporting on sex and gender	N/A
Reporting on race, ethnicity, or other socially relevant groupings	N/A
Population characteristics	N/A
Recruitment	N/A
Ethics oversight	N/A

Note that full information on the approval of the study protocol must also be provided in the manuscript.

Field-specific reporting

Please select the one below that is the best fit for your research. If you are not sure, read the appropriate sections before making your selection.

☒ Life sciences ☐ Behavioural & social sciences ☐ Ecological, evolutionary & environmental sciences

For a reference copy of the document with all sections, see nature.com/documents/nr-reporting-summary-flat.pdf

Life sciences study design

All studies must disclose on these points even when the disclosure is negative.

Sample size	Power calculations for determining the number of animals per group were based on effect sizes observed in our previous publications and were designed to detect a minimum difference of greater than 15% between experimental groups with sufficient statistical power (typically 80% or higher). Based on these calculations, a standard group size of n = 6 mice was generally used for most experiments, unless otherwise specified.<br><br>All animals enrolled in the study were included in the final analysis, and no exclusions were made based on outcomes or technical issues. To minimize potential bias, experiments were conducted in a blinded fashion whenever possible. This included blinding of investigators during sample processing, data acquisition, and analysis. For example, histological assessments were performed by a pathologist blinded to the experimental conditions, and data files were coded before analysis and only decoded after statistical evaluation was completed.

	This rigorous approach to sample size determination, inclusion criteria, and blinding was applied consistently across experiments to ensure the reliability, reproducibility, and transparency of the results.
Data exclusions	No animals were excluded from the study
Replication	All experiments were conducted with 2–3 independent biological replicates, and data from these replicates were pooled for analysis to ensure robustness and reproducibility of findings. Where appropriate, studies were analyzed and stratified by sex, with results graphed separately for males and females to identify potential sex-specific effects. This approach allowed for both combined and gender-specific interpretations of the data, contributing to a more comprehensive understanding of the biological outcomes.
Randomization	Animals were randomly assigned to experimental groups using a computer-based randomization tool (www.randomizer.org) to ensure unbiased group allocation. Randomization was performed prior to the start of treatment or intervention, and all investigators involved in data collection were blinded to group assignments whenever possible. Group sizes were balanced to account for potential confounding variables such as sex, age, and body weight, and cage placement was randomized to minimize environmental bias. These procedures were implemented to promote rigor, reproducibility, and transparency in the experimental design.
Blinding	Whenever possible, experiments were conducted in a blinded fashion to minimize bias. Samples were coded prior to analysis and only decoded after data collection and interpretation were completed. For example, histological assessments were scored independently by a pathologist who was blinded to the experimental groups. Similarly, in behavioral and biochemical assays, investigators analyzing the data were not involved in the experimental setup or treatment assignments. In studies involving image quantification or cell counting, images were randomized and analyzed using predefined criteria without knowledge of the group identity. These blinding procedures were implemented consistently to ensure objectivity and reproducibility of the findings.

Reporting for specific materials, systems and methods

We require information from authors about some types of materials, experimental systems and methods used in many studies. Here, indicate whether each material, system or method listed is relevant to your study. If you are not sure if a list item applies to your research, read the appropriate section before selecting a response.

Materials & experimental systems

n/a	Involved in the study	
☐	☒	Antibodies
☐	☒	Eukaryotic cell lines
☐	☐	Palaeontology and archaeology
☐	☒	Animals and other organisms
☐	☐	Clinical data
☐	☐	Dual use research of concern
☐	☐	Plants

Methods

n/a	Involved in the study	
☐	☐	ChIP-seq
☐	☐	Flow cytometry
☐	☐	MRI-based neuroimaging

Antibodies

Antibodies used

Primary Antibodies (used at 1:1000 dilution in TTBS):
Ketohexokinase (KHK)
Vendor: Sigma
Catalog #: HPA007040
RRID: AB_1079185
Alcohol Dehydrogenase 1 (ADH1)
Vendor: Cell Signaling Technology
Catalog #: 5295
RRID: AB_10626624
Aldehyde Dehydrogenase 1A1 (ALDH1A1)
Vendor: Cell Signaling Technology
Catalog #: 12035
RRID: AB_2797805
Aldehyde Dehydrogenase 2 (ALDH2)
Vendor: Cell Signaling Technology
Catalog #: 18818
RRID: AB_2798804
Acetyl-CoA Synthetase 1 (AceCS1)
Vendor: Cell Signaling Technology
Catalog #: 3658
RRID: AB_2222710
Fatty Acid Synthase (FAS)
Vendor: Cell Signaling Technology
Catalog #: 4233
RRID: AB_2100359
Acetyl-CoA Carboxylase (ACC)
Vendor: Cell Signaling Technology
Catalog #: 3676

RRID: AB_2219397
ATP Citrate Lyase (ACL)
Vendor: Cell Signaling Technology
Catalog #: 4332
RRID: AB_2223744
ΔFosB
Vendor: Cell Signaling Technology
Catalog #: 14695
RRID: AB_2798577
β-Actin
Vendor: Cell Signaling Technology
Catalog #: 4968
RRID: AB_2313904
Secondary Antibodies (used at 1:2000 dilution in TTBS):
Anti-Rabbit IgG, HRP-linked Antibody
Vendor: Cell Signaling Technology
Catalog #: 7074
RRID: AB_2099233
Anti-Mouse IgG, HRP-linked Antibody
Vendor: Cell Signaling Technology
Catalog #: 7076
RRID: AB_330924

Validation

All antibodies used in this study were commercially validated by the manufacturers, primarily Cell Signaling Technology, which provides extensive validation data, including specificity, sensitivity, and application suitability. In addition to manufacturer validation, the majority of these antibodies have been previously validated and successfully used in our own published studies, confirming their performance in relevant tissues and applications (e.g., Western blotting).

Specifically, antibodies such as Ketohexokinase (KHK) were further validated in-house using tissue samples from our KHK-A/C knockout mouse models, in which the absence of the target protein served as a negative control and confirmed antibody specificity. This approach ensures the reliability and accuracy of detection for both wild-type and genetically modified samples.

Together, the use of manufacturer-validated antibodies and experimental confirmation through genetically engineered models provides strong assurance of antibody specificity and suitability for the reported findings.

Eukaryotic cell lines

Policy information about cell lines and Sex and Gender in Research

Cell line source(s)

The GLUTag cell line with catalog number SCC652 is available from MilliporeSigma.it is derived from a murine enteroendocrine L-cell line originally established by Dr. Patricia Brubaker, University of Toronto)

Authentication

The GLUTag cell line was obtained directly from the vendor and was accompanied by the manufacturer's documentation confirming its identity and origin. GLUTag is a well-characterized murine enteroendocrine L-cell line originally established by Dr. Patricia Brubaker (University of Toronto) and is widely used for studies involving glucagon-like peptide-1 (GLP-1) secretion and nutrient sensing.

The identity and functional characteristics of the GLUTag cells were confirmed based on morphology, growth behavior, and hormone secretion profile (e.g., GLP-1 release in response to nutrient stimulation), in line with previously published data. Cells were maintained under standardized conditions, used at low passage numbers, and routinely monitored for consistency in response and phenotype.

Mycoplasma contamination

Cell line was tested negative for Mycoplasma

Commonly misidentified lines
(See ICLAC register)

Name any commonly misidentified cell lines used in the study and provide a rationale for their use.

Palaeontology and Archaeology

Specimen provenance

N/A

Specimen deposition

Indicate where the specimens have been deposited to permit free access by other researchers.

Dating methods

If new dates are provided, describe how they were obtained (e.g. collection, storage, sample pretreatment and measurement), where they were obtained (i.e. lab name), the calibration program and the protocol for quality assurance OR state that no new dates are provided.

☐ Tick this box to confirm that the raw and calibrated dates are available in the paper or in Supplementary Information.

Ethics oversight	*Identify the organization(s) that approved or provided guidance on the study protocol, OR state that no ethical approval or guidance was required and explain why not.*

Note that full information on the approval of the study protocol must also be provided in the manuscript.

Animals and other research organisms

Policy information about studies involving animals; ARRIVE guidelines recommended for reporting animal research, and Sex and Gender in Research

Laboratory animals	AR (Akr1b3) knockout mice were generated via CRISPR/Cas9 at the University of Colorado and previously characterized. KHK-A/C and KHK-A knockout mice, originally developed by David Bonthron (UK), have been backcrossed onto a C57Bl6 background for over 7 generations at the University of Colorado, where conditional KHK floxed mice (KHKFl/Fl) were also created; additionally, Crossed High Ethanol Preferring (cHAP) mice were obtained from selective breeding of HAP1 and HAP2 lines.
Wild animals	N/A
Reporting on sex	The majority of studies were conducted in both male and female. Data analysis was separated by sex in order to identify potential sex difference sin our study.
Field-collected samples	N/A
Ethics oversight	All animal experiments were performed in accordance with the NIH Guide for the Care and Use of Laboratory Animals. The study protocol was approved by the Institutional Animal Care and Use Committee (IACUC) of the University of Colorado (Aurora, CO) under protocol number 1253 (PI: Lanaspa).

Note that full information on the approval of the study protocol must also be provided in the manuscript.

Clinical data

Policy information about clinical studies

All manuscripts should comply with the ICMJE guidelines for publication of clinical research and a completed CONSORT checklist must be included with all submissions.

Clinical trial registration	N/A
Study protocol	N/A
Data collection	N/A
Outcomes	N/A

Dual use research of concern

Policy information about dual use research of concern

Hazards

Could the accidental, deliberate or reckless misuse of agents or technologies generated in the work, or the application of information presented in the manuscript, pose a threat to:

No	Yes	
☒	☐	Public health
☒	☐	National security
☒	☐	Crops and/or livestock
☒	☐	Ecosystems
☒	☐	Any other significant area

Experiments of concern

Does the work involve any of these experiments of concern:

No	Yes	
☒	☐	Demonstrate how to render a vaccine ineffective
☒	☐	Confer resistance to therapeutically useful antibiotics or antiviral agents
☒	☐	Enhance the virulence of a pathogen or render a nonpathogen virulent
☒	☐	Increase transmissibility of a pathogen
☒	☐	Alter the host range of a pathogen
☒	☐	Enable evasion of diagnostic/detection modalities
☒	☐	Enable the weaponization of a biological agent or toxin
☒	☐	Any other potentially harmful combination of experiments and agents

Plants

Seed stocks	N/A
Novel plant genotypes	N/A
Authentication	N/A

ChIP-seq

Data deposition

☐ Confirm that both raw and final processed data have been deposited in a public database such as GEO.

☐ Confirm that you have deposited or provided access to graph files (e.g. BED files) for the called peaks.

Data access links<br>*May remain private before publication.*	N/A
Files in database submission	N/A
Genome browser session<br>(e.g. UCSC)	N/A

Methodology

Replicates	N/A
Sequencing depth	N/A
Antibodies	N/A
Peak calling parameters	N/A
Data quality	N/A
Software	N/A

Flow Cytometry

Plots

Confirm that:

- [ ] The axis labels state the marker and fluorochrome used (e.g. CD4-FITC).
- [ ] The axis scales are clearly visible. Include numbers along axes only for bottom left plot of group (a 'group' is an analysis of identical markers).
- [ ] All plots are contour plots with outliers or pseudocolor plots.
- [ ] A numerical value for number of cells or percentage (with statistics) is provided.

Methodology

Sample preparation	N/A
Instrument	N/A
Software	N/A
Cell population abundance	N/A
Gating strategy	N/A

- [ ] Tick this box to confirm that a figure exemplifying the gating strategy is provided in the Supplementary Information.

Magnetic resonance imaging

Experimental design

Design type	N/A
Design specifications	N/A
Behavioral performance measures	N/A

Acquisition

Imaging type(s)	N/A
Field strength	N/A
Sequence & imaging parameters	N/A
Area of acquisition	N/A

Diffusion MRI ☐ Used ☐ Not used

Preprocessing

Preprocessing software	N/A
Normalization	N/A
Normalization template	N/A
Noise and artifact removal	N/A
Volume censoring	*Define your software and/or method and criteria for volume censoring, and state the extent of such censoring.*

Statistical modeling & inference

Model type and settings	N/A
Effect(s) tested	N/A

Specify type of analysis: ☐ Whole brain ☐ ROI-based ☐ Both

Statistic type for inference

(See Eklund et al. 2016)

Specify voxel-wise or cluster-wise and report all relevant parameters for cluster-wise methods.

Correction

N/A

Models & analysis

n/a	Involved in the study
☐	☐ Functional and/or effective connectivity
☐	☐ Graph analysis
☐	☐ Multivariate modeling or predictive analysis

Functional and/or effective connectivity

N/A

Graph analysis

N/A

Multivariate modeling and predictive analysis

Specify independent variables, features extraction and dimension reduction, model, training and evaluation metrics.
