# Supplementary material for: Identification of a common ketohexokinase-dependent link driving alcohol intake and alcohol-associated liver disease in mice
Source: Nat Metab. 2025 Nov 10;7(11):2250–67. doi: 10.1038/s42255-025-01402-x (PMC12638252; doi:10.1038/s42255-025-01402-x)

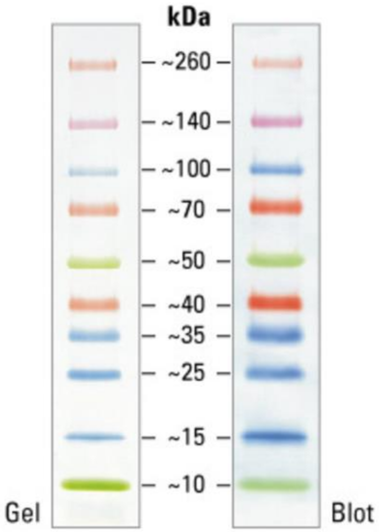

Spectra Multicolor Broad Range Ladder (#26634, Thermo Fisher)

Figure 1N dFosB

Figure 1N stripped and reprobed for GAPDH

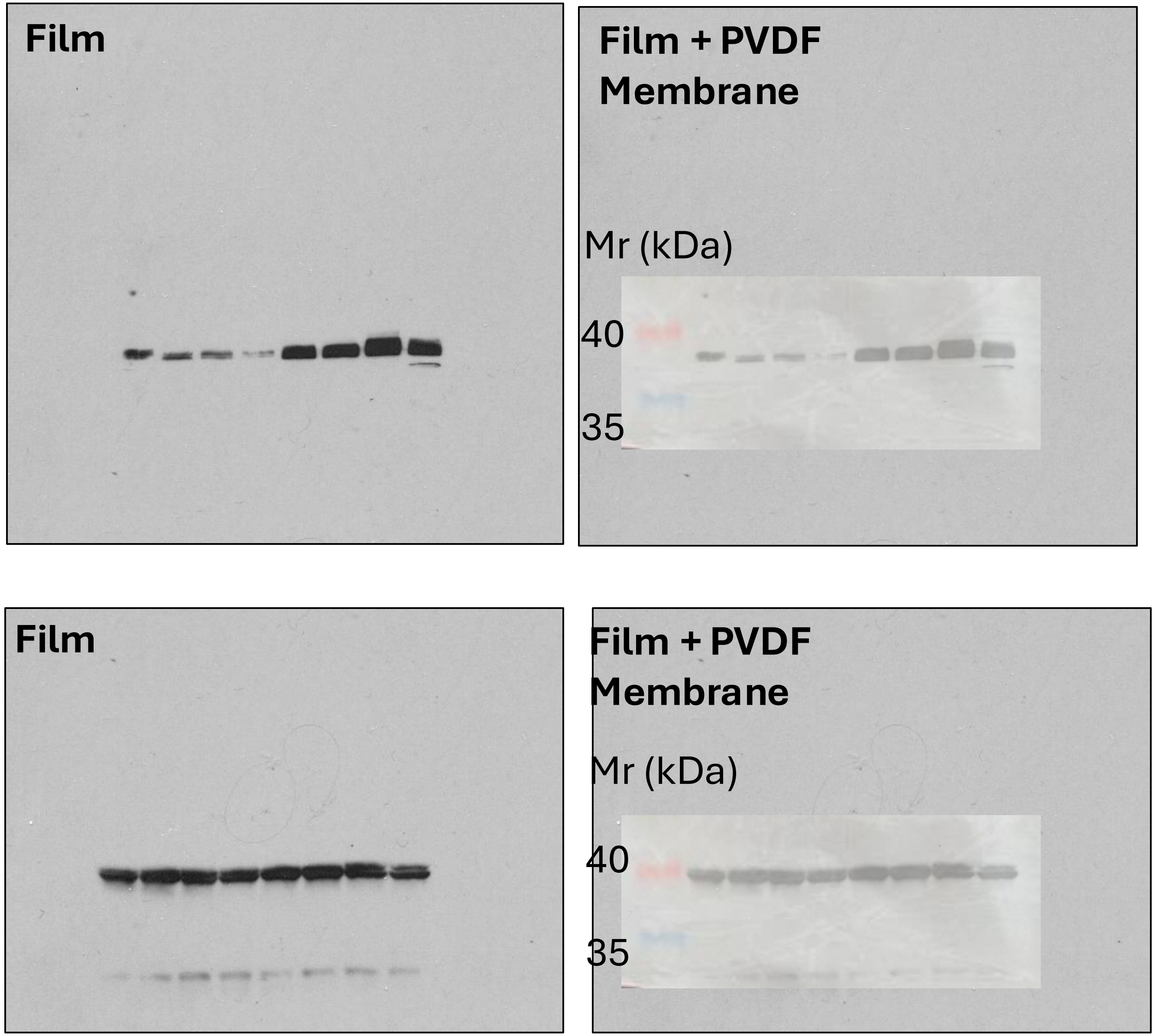

Figure 1O
KHK-A/C, Liver

Film

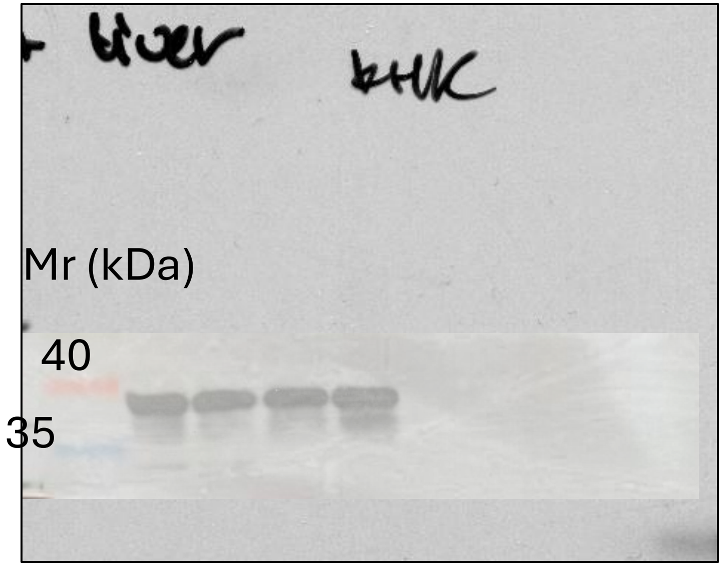

Film + PVDF Membrane

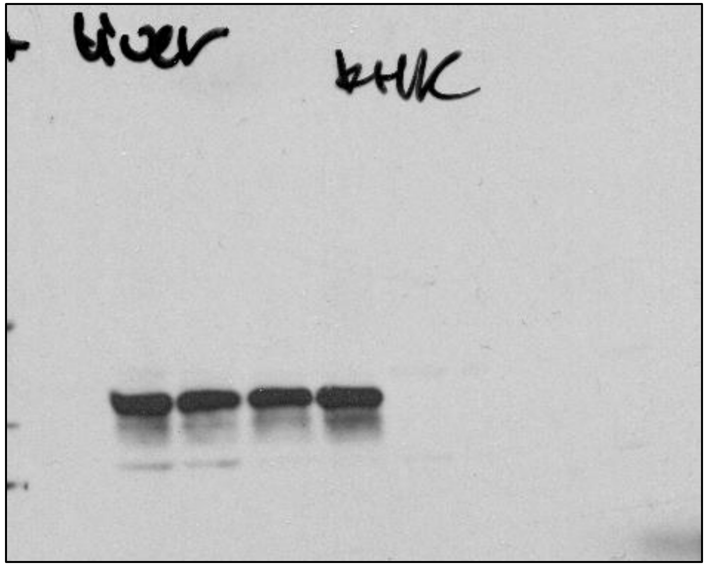

Figure 1N dFosB NAcc stripped and reprobed for GAPDH

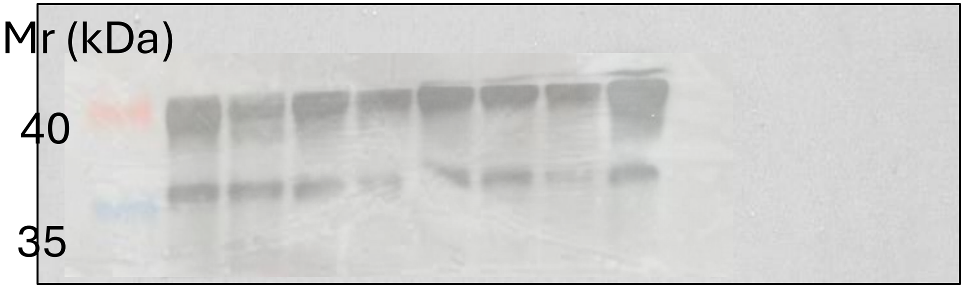

dFosB, NAcc
Film

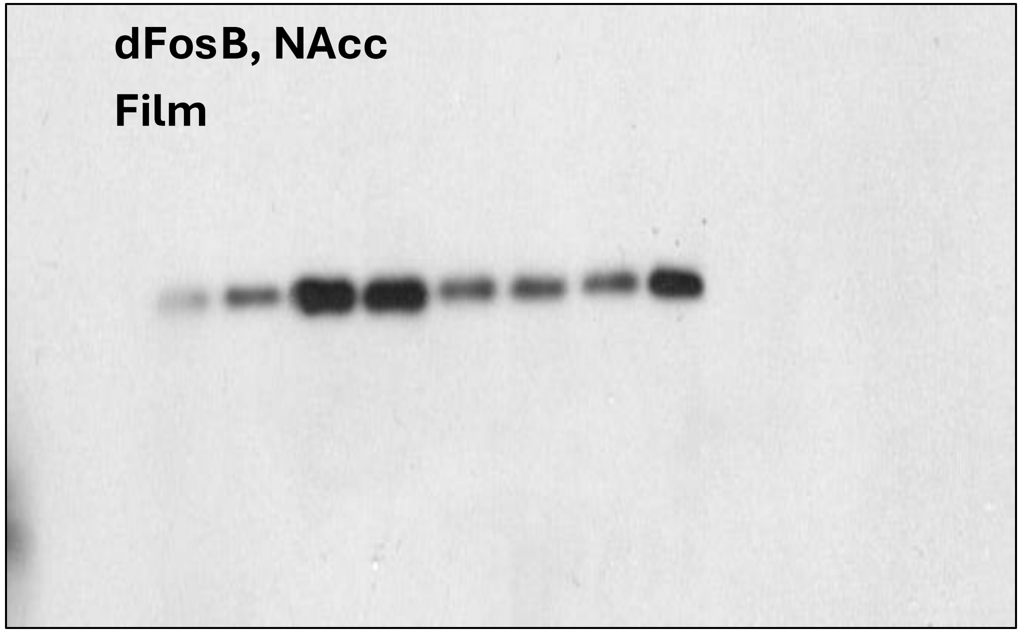

Film + PVDF Membrane

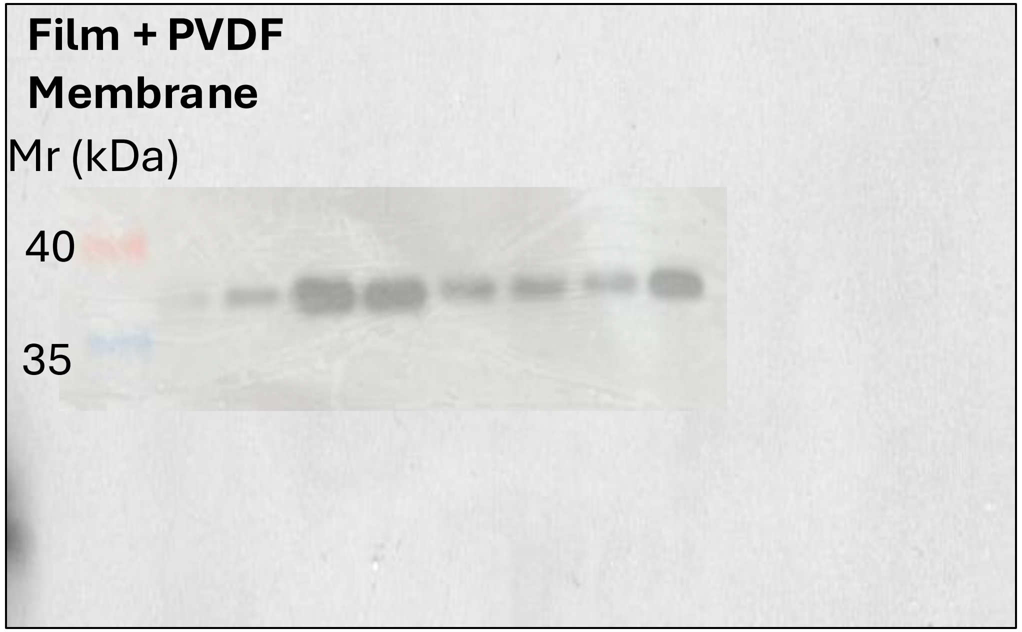

Figure 2C

AR

Film

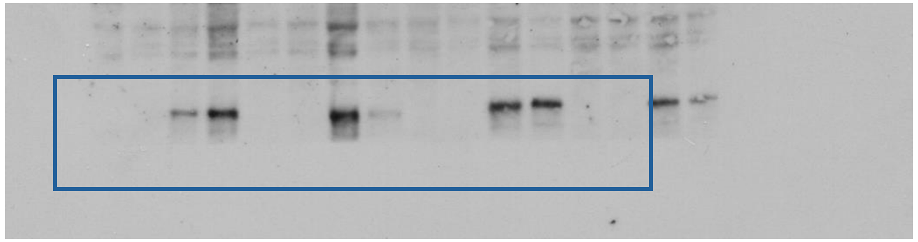

Film + PVDF Membrane

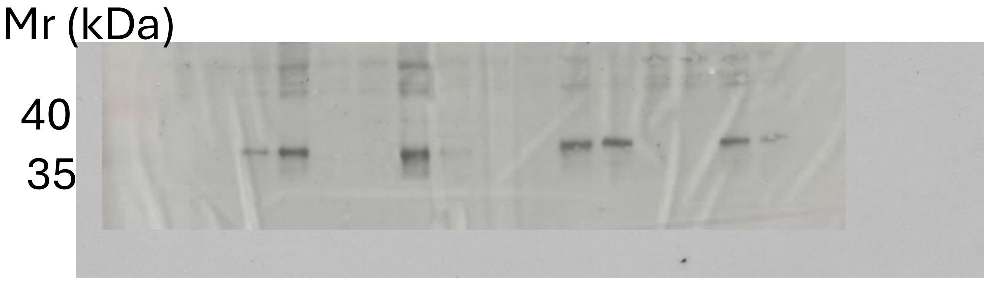

Vinculin

Film, stripped and reprobed for Vinculin

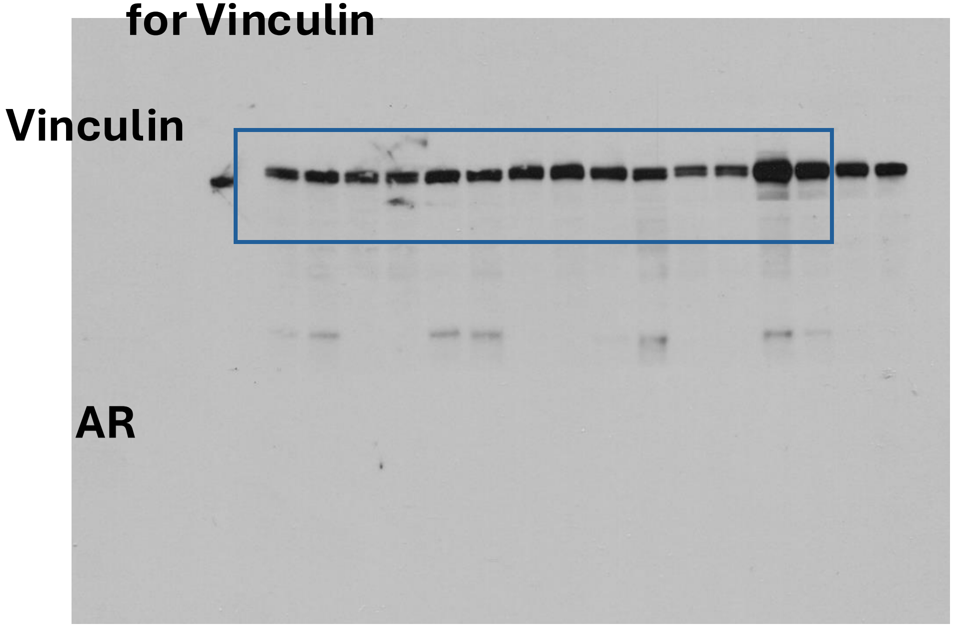

Film + PVDF Membrane

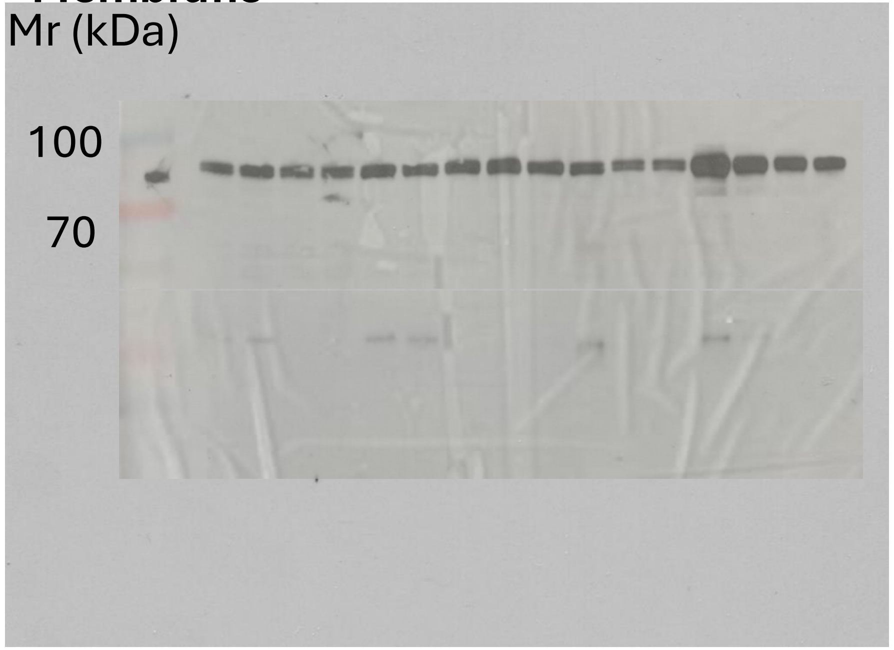

Figure 2F

AR

Film

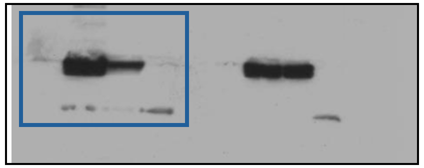

Film + PVDF Membrane

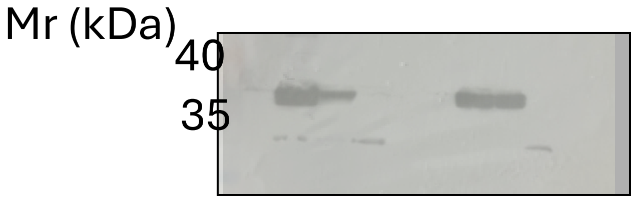

Actin, Stripped and reprobed from ADH

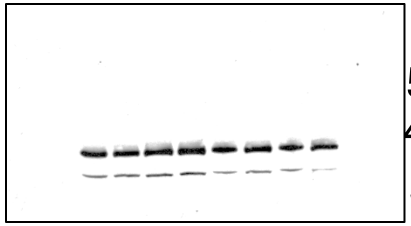

Film + PVDF Membrane

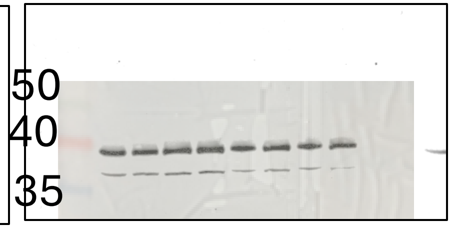

Figure 3H

ADH

Film

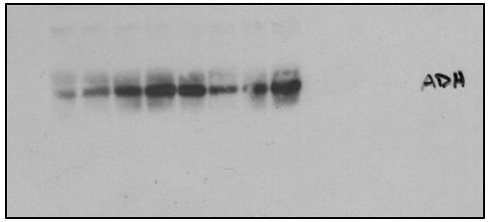

Film + PVDF Membrane

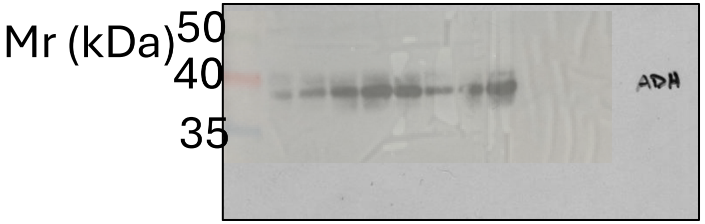

ALDH1A1

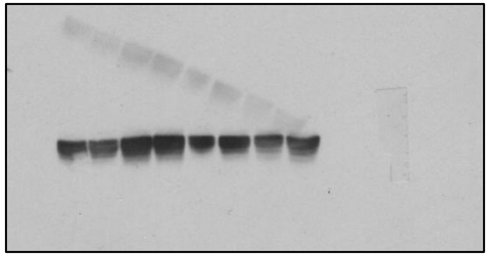

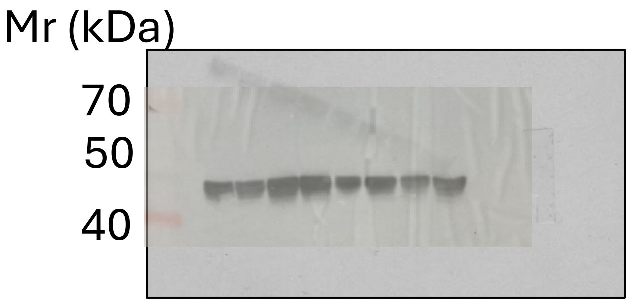

ALDH2

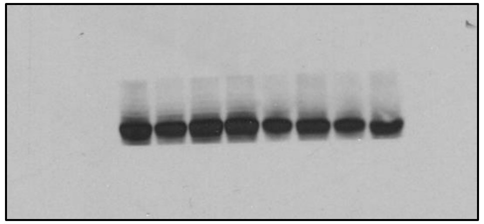

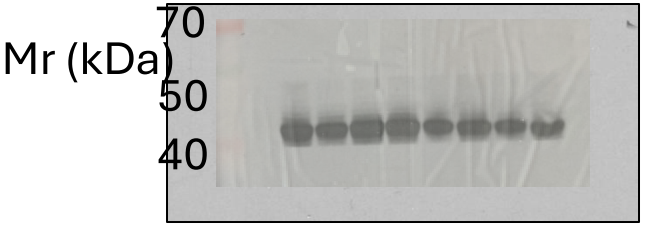

AceCS1

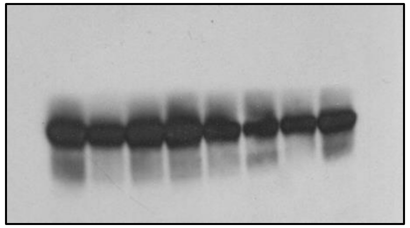

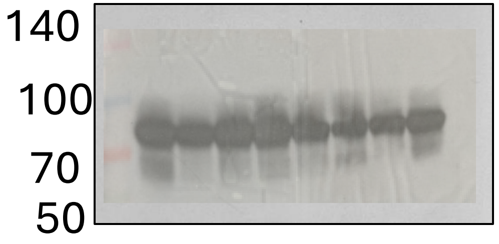

Figure 3M

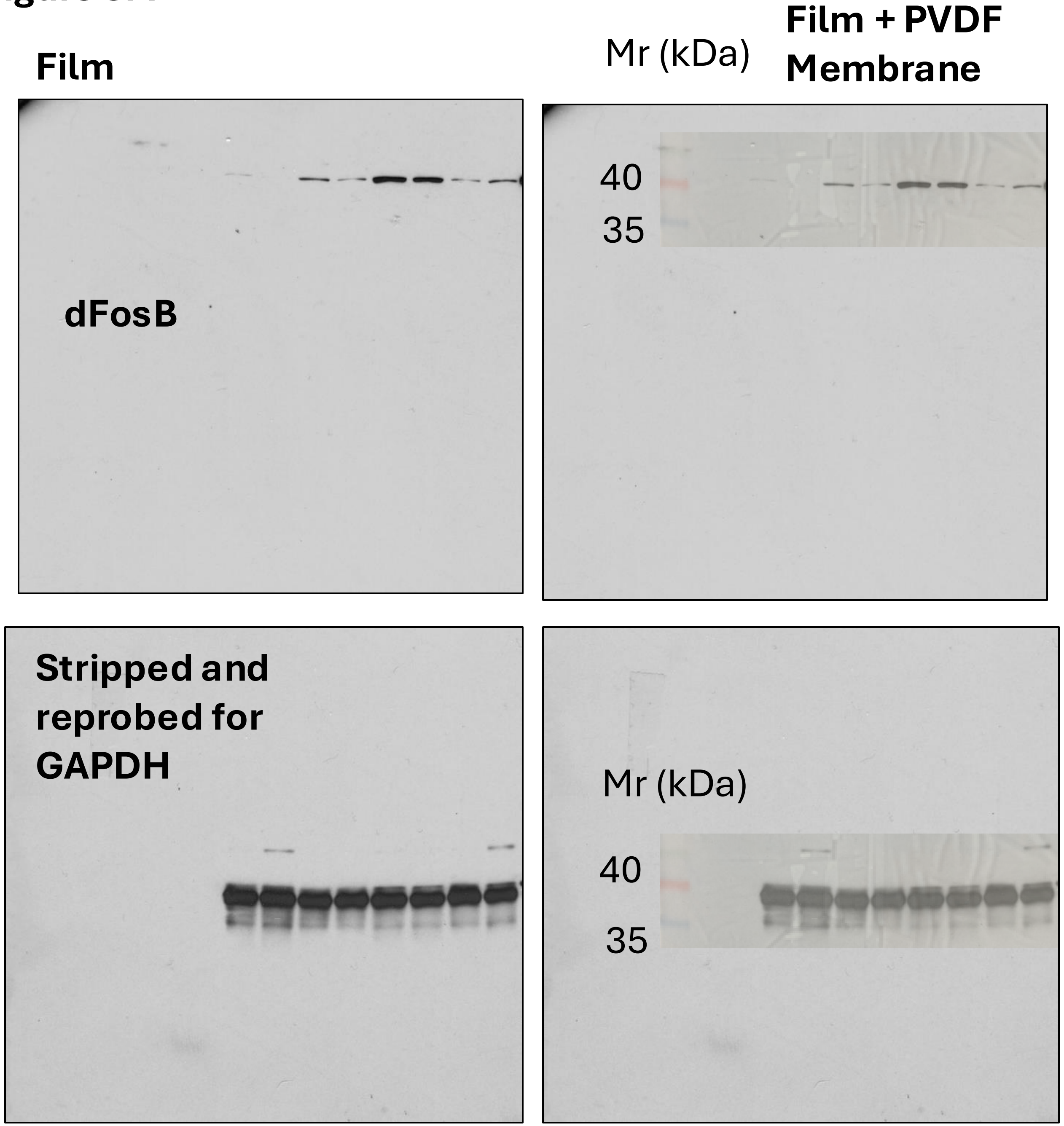
Film
Mr (kDa)
Film + PVDF Membrane
40
35
dFosB
Stripped and reprobed for GAPDH
Mr (kDa)
40
35

Figure 4A

AR

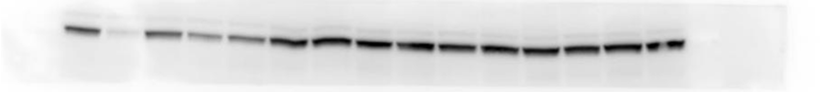

Mr (kDa)

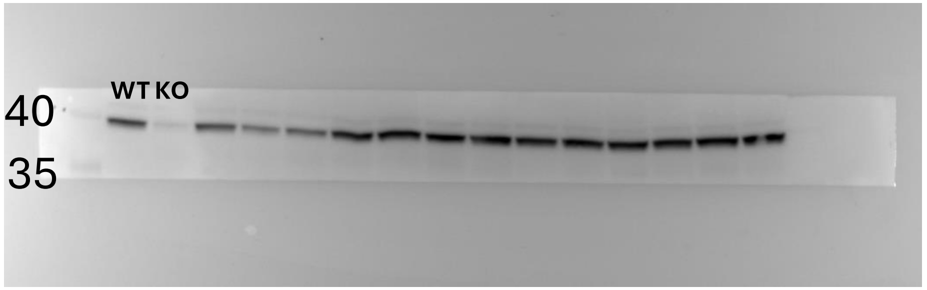

KHK-A/C

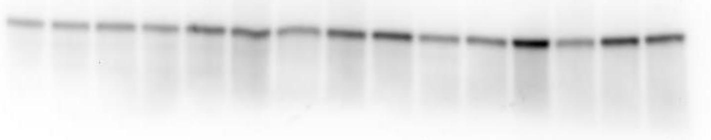

Mr (kDa)

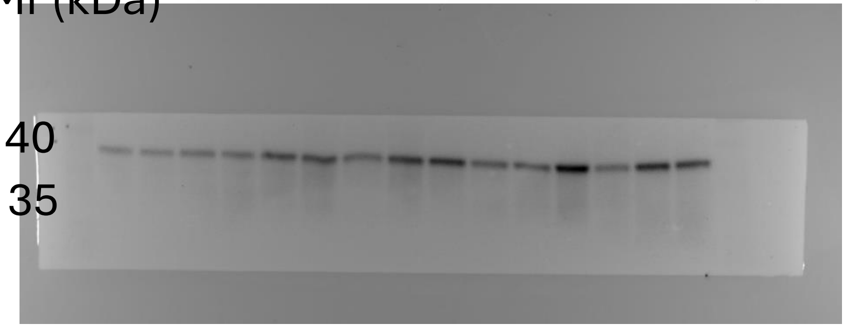

SDH

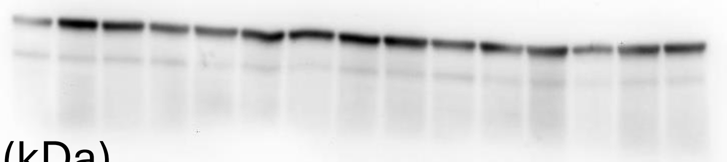

Mr (kDa)

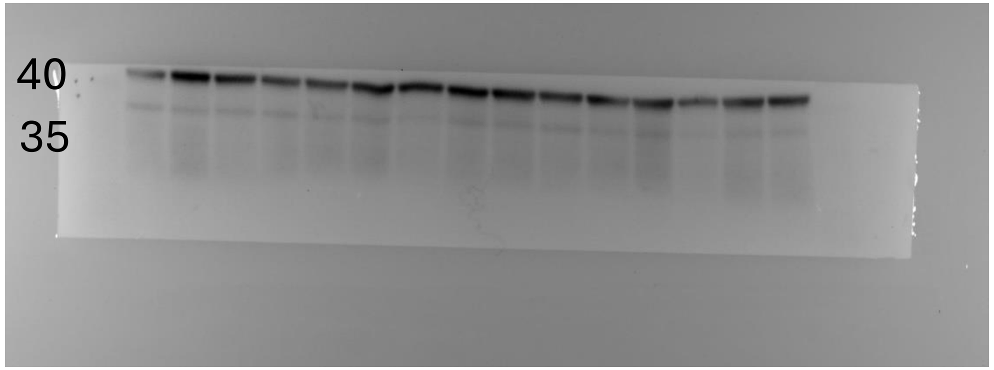

Figure 5E

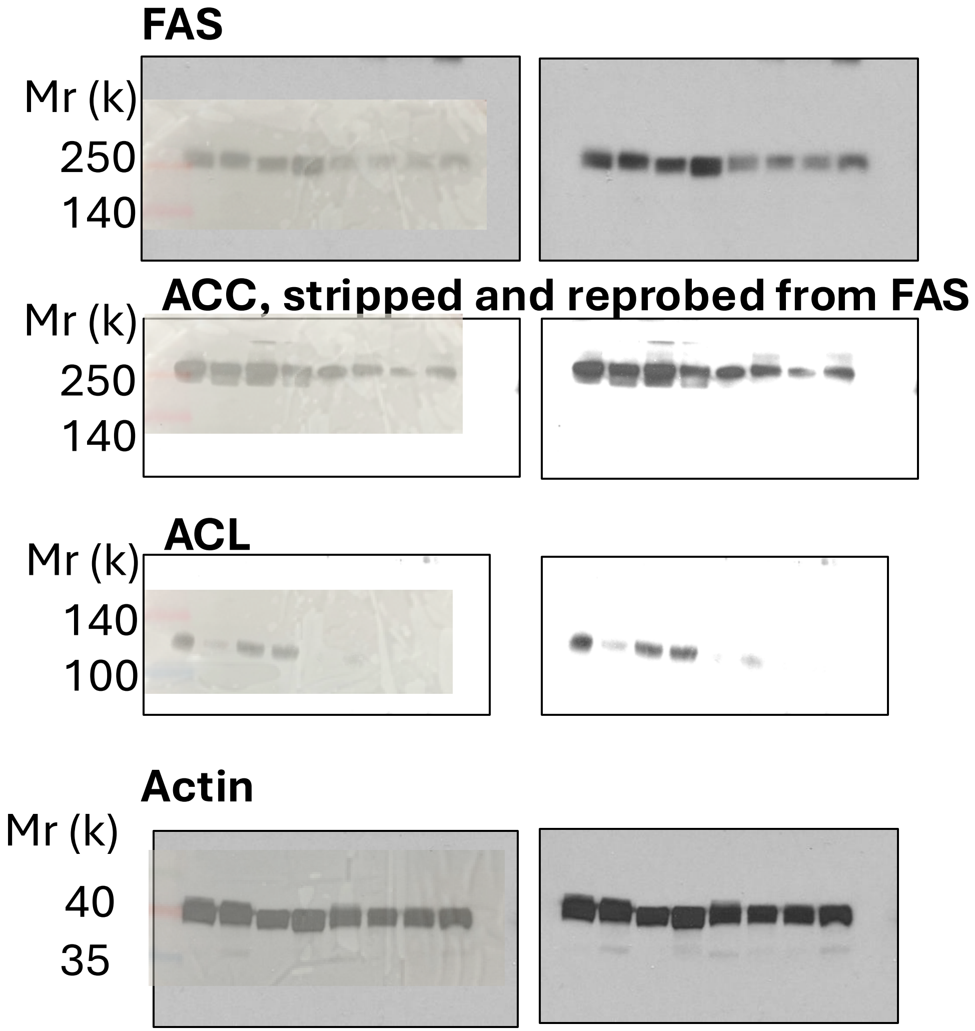

Figure 6A

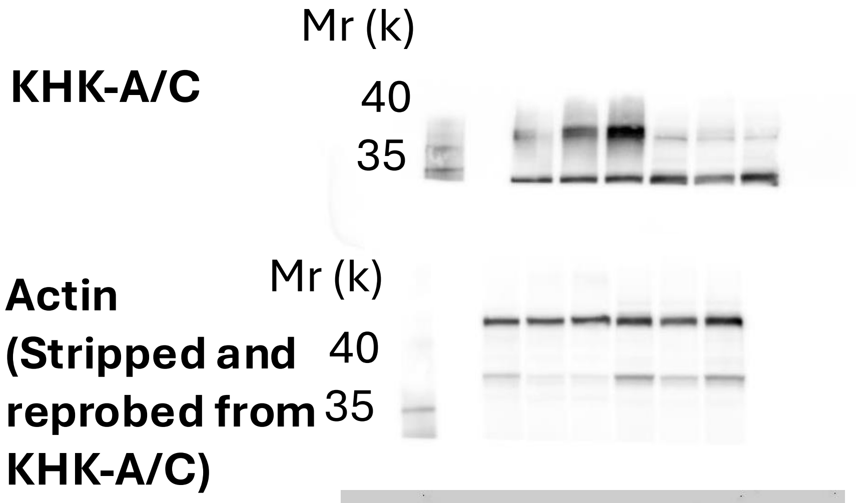

Figure 6E

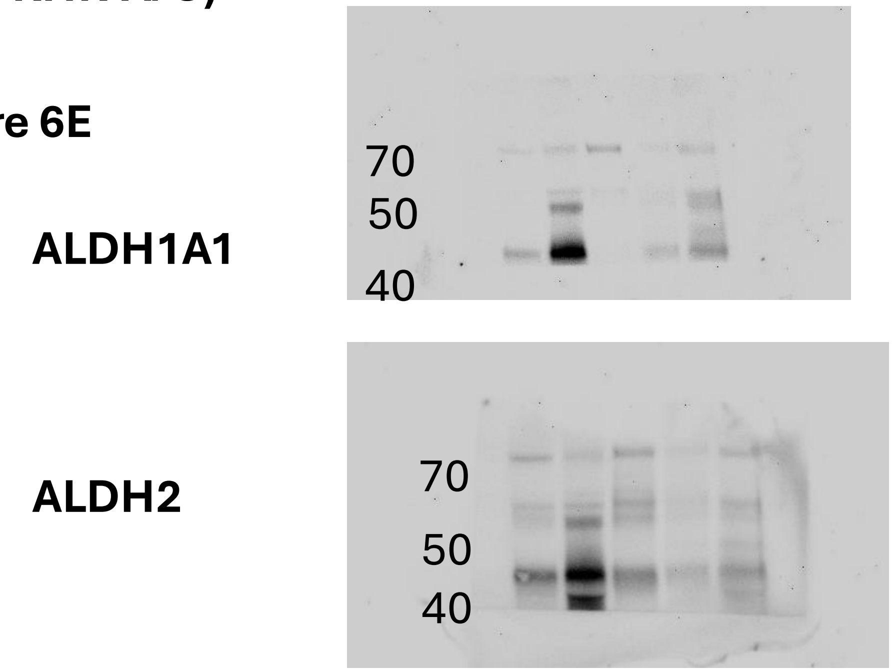

Extended Data 3E

KHK-A/C

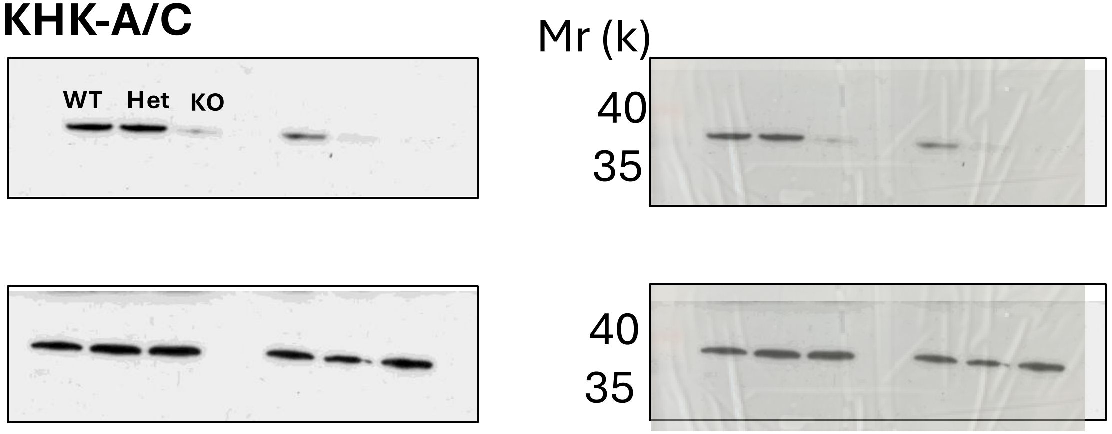

Actin (reprobed from KHK-A/C)

Extended Data 4A

Glut5

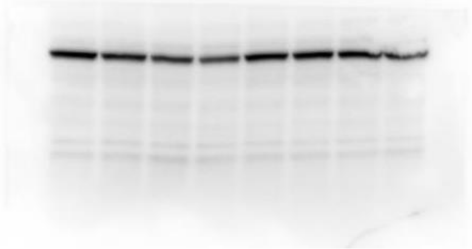

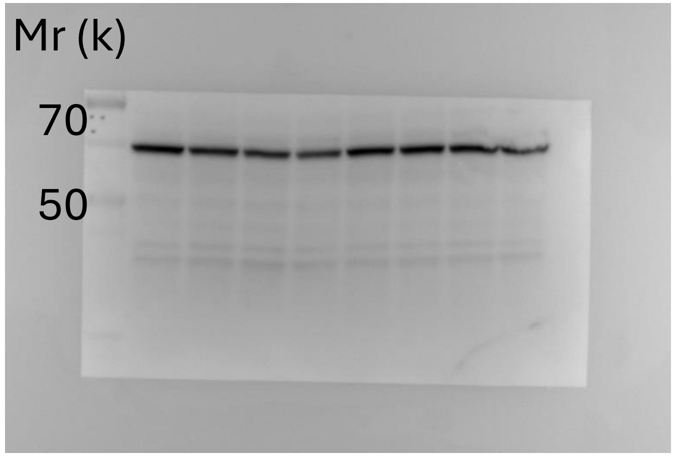

KHK-A/C

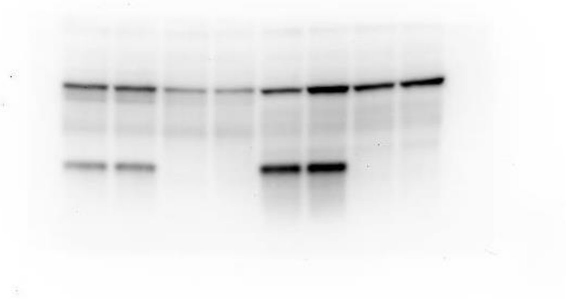

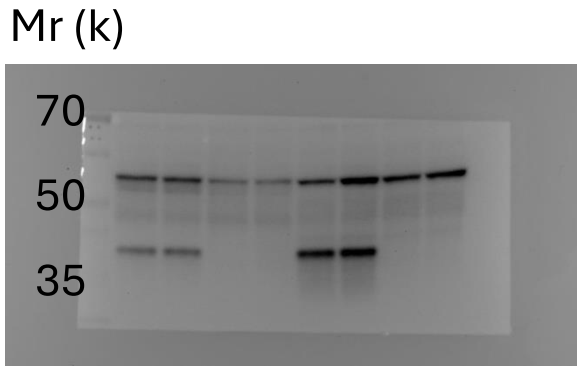

Cyp2E1

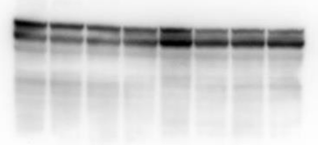

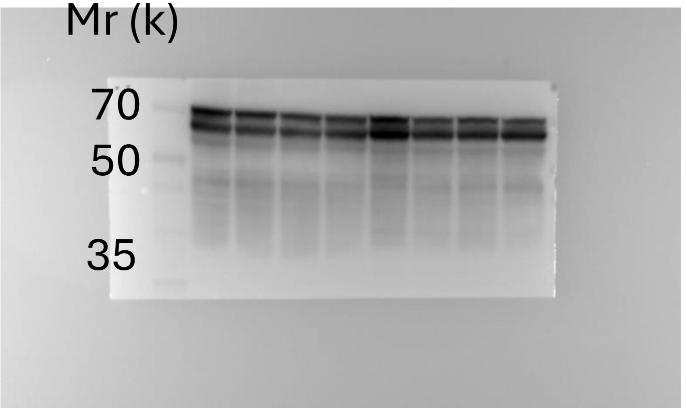

Vinculin

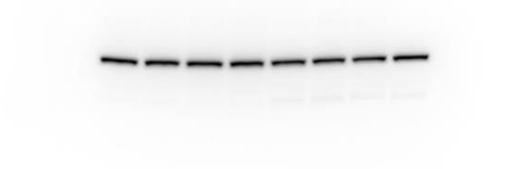

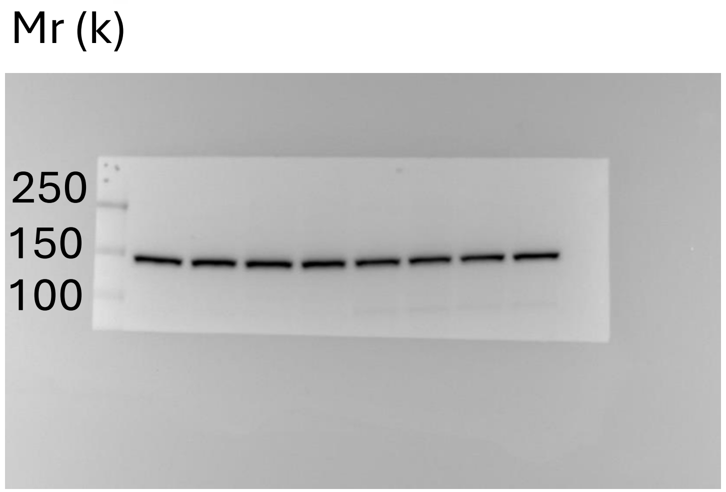

Extended Data 4B

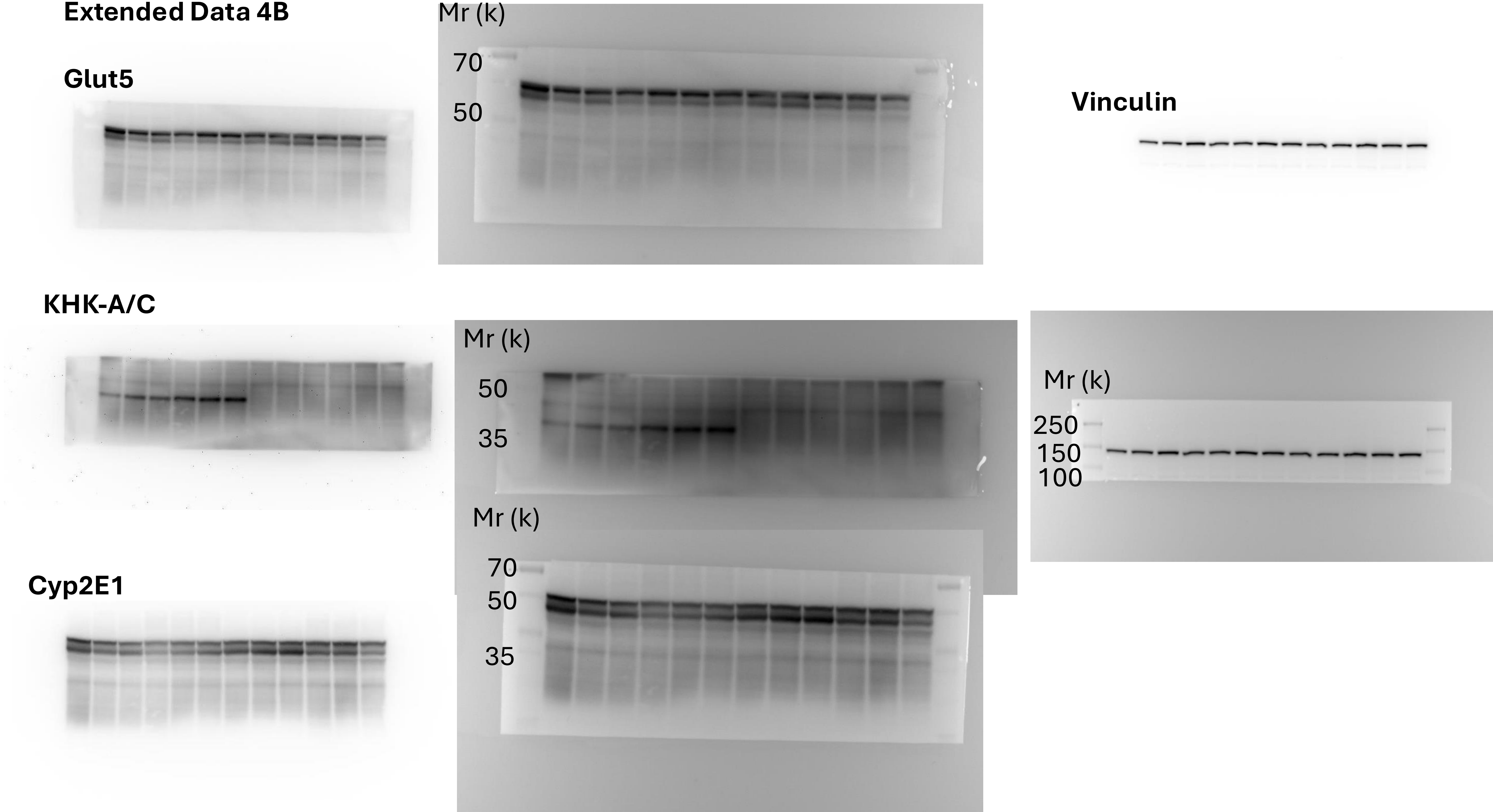

Supplement: Supplementary file 3 — Unprocessed western blots with size markers. [file 42255_2025_1402_MOESM3_ESM.pdf]
